# Deepdefense: annotation of immune systems in prokaryotes using deep learning

**DOI:** 10.1093/gigascience/giae062

**Published:** 2024-10-10

**Authors:** Sven Hauns, Omer S Alkhnbashi, Rolf Backofen

**Affiliations:** Bioinformatics Group, Department of Computer Science, University of Freiburg, Freiburg 79110, Germany; Center for Applied and Translational Genomics (CATG), Mohammed Bin Rashid University of Medicine and Health Sciences, Dubai Healthcare City, Al Razi St. P.O 505055, Dubai, United Arab Emirates; College of Medicine, Mohammed Bin Rashid University of Medicine and Health Sciences, Dubai Healthcare City, Al Razi St. 505055, Dubai, United Arab Emirates; Bioinformatics Group, Department of Computer Science, University of Freiburg, Freiburg 79110, Germany; Signalling Research Centres BIOSS and CIBSS, University of Freiburg, Freiburg 79104, Germany

**Keywords:** immune systems, deep learning, classification, genome

## Abstract

**Background:**

Due to a constant evolutionary arms race, archaea and bacteria have evolved an abundance and diversity of immune responses to protect themselves against phages. Since the discovery and application of CRISPR-Cas adaptive immune systems, numerous novel candidates for immune systems have been identified. Previous approaches to identifying these new immune systems rely on hidden Markov model (HMM)–based homolog searches or use labor-intensive and costly wet-lab experiments. To aid in finding and classifying immune systems genomes, we use machine learning to classify already known immune system proteins and discover potential candidates in the genome. Neural networks have shown promising results in classifying and predicting protein functionality in recent years. However, these methods often operate under the closed-world assumption, where it is presumed that all potential outcomes or classes are already known and included in the training dataset. This assumption does not always hold true in real-world scenarios, such as in genomics, where new samples can emerge that were not previously accounted for in the training phase.

**Results:**

In this work, we explore neural networks for immune protein classification, deal with different methods for rejecting unrelated proteins in a genome-wide search, and establish a benchmark. Then, we optimize our approach for accuracy. Based on this, we develop an algorithm called Deepdefense to predict immune cassette classes based on a genome. This design facilitates the differentiation between immune system–related and unrelated proteins by analyzing variations in model-predicted confidence values, aiding in the identification of both known and potentially novel immune system proteins. Finally, we test our approach for detecting immune systems in the genome against an HMM-based method.

**Conclusions:**

Deepdefense can automatically detect genes and define cassette annotations and classifications using 2 model classifications. This is achieved by creating an optimized deep learning model to annotate immune systems, in combination with calibration methods, and a second model to enable the scanning of an entire genome.

**Key Points**

- We develop a deep learning–based approach for the classification of Doron immune systems in whole genomes based on 2 classifiers.
- The first classifier filters proteins most likely not belonging to the immune system, while the second classifier classifies immune system subclasses or rejects unrelated data.
- The resulting output classification is then used to build specific cassettes for each immune system type.
- We improve the model calibration using different methods to reject unrelated data reliably.
- Using the calibrated model, we also suggest candidates that may constitute a new immune system.

## Introduction

Viruses are the most abundant biological entities in biospheres such as soil and sea, vastly outnumbering prokaryotes [1–4]. Given such an abundance of numbers, phages and plasmids frequently attack bacteria and archaea. Because of such attacks and the resulting arms race, they have evolved various techniques to defend themselves [5, 6].

These techniques can be grouped into 2 general defense mechanisms based on the principle of their action: (i) innate or adaptive immunity and (ii) programmed cell death and dormancy [7, 8]. Innate immunity is often based on restriction-modification (R-M) systems, where a restriction enzyme cuts unmethylated DNA. As the own DNA is methylated, this protects against any unmethylated foreign DNA [6]. In contrast, the CRISPR-Cas systems provide adaptive immunity, which recognizes and degrades specific viral nucleic acids [9]. The ability of the CRISPR systems to precisely cut and integrate viral nucleic acids into the bacteria genome has led to the creation of important new genetic tools, such as Cas9, for gene editing [10–12].

The success of CRISPR/Cas9 has fueled the search for new members of the CRISPR-Cas systems and led to many different computational approaches, both scoring or machine learning (ML) based, to identify new members of the CRISPR-Cas system [13–23]. However, no ML approach exists to date to identify immune systems different from CRISPR and to suggest candidates for further exploration.

We opt for deep learning, anticipating that its capacity for automatically generating intricate features will enable the identification of proteins, even those with lower structural similarity to known samples. The ability of deep learning models to recognize and leverage subtle patterns for classification should empower it to capture and comprehend complex relationships, thereby enhancing their effectiveness in annotation.

To set up such an ML approach for non-CRISPR immune systems, we have to ask ourselves whether there is some chance to detect an immune system in some prokaryotic genome. The estimated proportion of genes coding for immune systems varies from about 0.0001% to 10% [24]. The given distributions for each defense system show the lower limit because many more variants need to be identified due to the rapid mutation of defense genes. The number of defense genes in a genome shows a linear scaling with the size of the genome [8]. The genomes responsible for immune response are often close together and form so-called defense islands [25]. In the following, we would like to exploit this property to predict immune cassettes based on gene predictions ultimately. Our approach focuses on identifying immune systems identified in [26], named Druantia, Gabija, Hachiman, Kiwa, Lamassu, Septu, Shedu, Thoeris, Wadjet, and Zorya. Following [27], these systems are called the “Doron systems.”

In this work, we present a deep learning approach called Deepdefense, designed to capture much of the relevant information used in manual annotation. It is purely based on features for different protein sequences related to immune systems. The proposed approach addresses the problem of annotating and classifying Doron defense gene systems into types and subtypes by using evidence from a series of consecutive genes that form part of a cassette to identify the various proteins involved. This approach enables us to represent the genomics of antiviral systems as cassettes of adjacent proteins encoded in the genome. As our features for the deep learning approach correspond to evidence for immune-related protein sequences, we can determine proteins whose evidence is critical for predicting types and subtypes. Since we want to use our tool to scan the entire genome, we need to be able to reject unrelated proteins reliably. We try to improve the problems caused by this closed-world assumption [28] by using techniques designed to improve model calibration. Furthermore, we compare this approach with the recently published approach based on the hidden Markov model (HMM) homology search [27] and demonstrate that Deepdefense can identify more immune systems in prokaryotic genomes. Additionally, the tool identifies potential new immune systems related to the Doron systems.

## Data Description

Our dataset comprises 21,196 unique validated samples [26]. To ensure ample training data for subclasses with limited samples, we employ a stratified 10-fold cross-validation split, allocating 10$\%$ of the dataset for testing in each cross-validation step and another 10$\%$ of the remaining dataset as a validation set for early stopping. Due to sampling in a stratified fashion, a representative number of samples for each class are contained in each test set. This results in 17,169 samples for training, 1,908 for validation, and 2,119 for testing in each cross-validation split. Additionally, we use BLAST to create datasets with varying degrees of sequence homology (95$\%$, 90$\%$, 80$\%$, 70$\%$, 60$\%$, 50$\%$, 40$\%$, and 30$\%$), and we also report our results on these sets.

The dataset we used was imbalanced for types, consisting of 1,263 Durantia samples, 3,723 Gajiba samples, 1,529 Hachiman samples, 6882 Wadjet samples, 637 Lamassu samples, 2807 Septu samples, 647 Shedu samples, 1,097 Thoeris samples, 745 Kiwa samples, and 1,866 Zorya samples. Hence, there were only roughly one-tenth of the samples of Lamassu than for Wadjet. We weighted the samples during training according to their distribution in the dataset to prevent overfitting to majority classes [29] by dividing the total number of samples by the number of samples in a class multiplied by the number of classes. Additionally, we used a dataset with 21,196 samples from bacteria and archaea that have an unrelated function to the immune system, also ensuring a low similarity to the known defense system by sequence homology and HMM-based search. In addition to cross-validation, we also used 10$\%$ of the data for an independent test dataset with a maximal sequence homology of 95. We created 12 different features characterizing the used proteins (e.g., protein length, instability index) as in [30]. The full list can be found in Supplementary Table S1.

## Materials and Methods

### Benchmarking uncertainty—calibration

In the past, neural networks were well calibrated to indicate useful certainties. Despite achieving much better accuracy today, this is only sometimes the case for modern neural networks [31]. Guo et al. [31] also observe that architecture decisions that increase accuracy can decrease calibration. Particular models with a high capacity using batch normalization, little weight decay, and cross-entropy loss tend to be miscalibrated.

Following the notation from  [32], we denote with *X* an instance in the features space $\mathcal {X}$. A supervised multiclass classification problem for *k* classes can be stated as finding a probabilistic classifier $\mathop {\hat{\mathbf {p}}}: \mathcal {X}\rightarrow \Delta _k$, yielding a probability vector $\mathop {\hat{\mathbf {p}}}(X) = (\hat{{p}}_{1}(X), ..., \hat{{p}}_{k}(X))^\top$ for the input $X\in \mathcal {X}$, where $\Delta _k = \lbrace (q_1 ... q_k)^\top \in [0,1]^k|\sum _{i=1}^k q_i = 1\rbrace$. In many other publications, the $i^{th}$ component $\hat{{p}}_{i}(X)$ of the probabilistic classifier is denoted as the conditional probability $p_\theta (Y=i\mid X)$, where $\theta$ are the parameters of the model.

Now in deep neuronal networks, it is common to define the probabilistic classifier via a softmax over the network’s *logits*  $\mathbf {z}(X) = (z_1(X), ...., z_k(X))^\top$ for a specific instance *X*. In this case, the predicted probability $\hat{{p}}_{i}(X)$ with $i \in \lbrace 1,...,k\rbrace$ is defined as


(1) \begin{eqnarray*}
\hat{{p}}_{i}(X) = \sigma _{SM}^{\mathbf {z}(X)}({z}_{i}(X)) =: \frac{\exp {({z}_{i}(X))}}{\sum _{j=1}^{k} \exp {(z_{j}(X)})}\
\end{eqnarray*}

In the following, we will write $\sigma _{SM}$ as short for $\sigma _{SM}^{\mathbf {z}(X)}$ when $\mathbf {z}$ is clear from the context. Furthermore, we will overload the symbol $\sigma _{SM}$ to also apply it to vectors. Using this, Eq. 1 can simply be written as $\mathop {\hat{\mathbf {p}}}(X) =\sigma _{SM}(\mathbf {z}(X))$. Now given any prediction vector $\mathbf {q}= (q_1 ... q_k)^\top \in \Delta _k$, the perfect multiclass calibration is then defined for a probabilistic classifier $\mathop {\hat{\mathbf {p}}}(X)$ as follows [32]:


(2) \begin{eqnarray*}
for\ i = 1, ..., k: \mathbb {P}(Y= i| \mathop {\hat{\mathbf {p}}}(X{})=\mathbf {q}) = q_i
\end{eqnarray*}

It simply states that for all instances *X* with the predicted class probabilities $\mathbf {q}$ (i.e., in the event $\mathop {\hat{\mathbf {p}}}(X)=\mathbf {q}$), the proportion of classes over these instances equals $\mathbf {q}$. Methods to improve the calibration were chosen to be ubiquitously usable and not rely on data augmentation. We decide not to use data augmentation techniques since they implicitly rely on the assumption that the input class is invariant to minor changes. We do not know whether this holds for our dataset. Hence, we test the methods *temperature, matrix*, and *vector* described in [31], as well as *Deep Open Classification (DOC)* from [33], *label smoothing* [34, 35], and *penalizing confidence* [36] in both single and ensemble use, as shortly described in the following subsections.

#### Cutoff

The most simplistic idea to identify uncertain class prediction is to create a cutoff for every class we predict.

#### Penalizing confidence

Now given a deep neuronal network where the probabilistic classifier $\mathop {\hat{\mathbf {p}}}(X)$ is defined using the softmax of the logits (Eq. 1), one problem that leads to miscalibration are overconfident predictions, which correspond to outputs favoring a particular class for each input *X*. This corresponds to probability distributions with low entropy. Thus, we can penalize confidence directly using the entropy [36]. The penalty is now activated by adding the entropy, given as


\begin{eqnarray*}
H(\mathop {\hat{\mathbf {p}}}(X{})) = - \sum _{i=1}^{k} \hat{{p}}_{i}(X)\log (\hat{{p}}_{i}(X)) \end{eqnarray*}

to the log-likelihood function during training,


\begin{eqnarray*}
L = - \sum _{X{} \in X}\sum _{i=1}^{k}(p_i \log (\hat{{p}}_{i}(X))) - \beta H(\mathop {\hat{\mathbf {p}}}(X)), \end{eqnarray*}

where $p_i$ is the empirical distribution of the $i^{th}$ label in the training set, and $\beta$ regulates the extent of punishment [36].

To avoid overfitting toward the end of the training process and still enable fast convergence, we can use a hinge loss that penalizes the confidence only when a certain entropy threshold is exceeded [36].


\begin{eqnarray*}
L = - \sum _{X{} \in X} \sum _{i=1}^{k}(p_i\log (\hat{{p}}_{i}(X))) - \beta \max (0, \Omega - H(\mathop {\hat{\mathbf {p}}}(X{}))) \end{eqnarray*}

$\Omega$ stands here for the entropy threshold that the output must exceed.

#### DOC

The DOC method describes the construction of a multiclass classifier with a 1-vs residual final layer of sigmoids. It uses Gaussian fitting to reduce the free space risk further. For the *i*^th^ sigmoid function with class *i*, the positive examples are all data points with $y = i$, and the negative examples are all data points with $y \ne i$. The output of the $i^{\text{th}}$ sigmoid on the input $X{}$ is then used as predicted probabilities (please note that the *predicted probabilities* in this case do not form a probability distribution as the probabilities do not sum up to 1; we are, however, following here the standard terms in the field as used in, e.g., [33]).


(3) \begin{eqnarray*}
\hat{{p}}_{i}(X) = Sigmoid(z_i(X)) \end{eqnarray*}

The loss of 1 sample $X{}$ is then the sum of all log-loss functions of all sigmoids [33]:


\begin{eqnarray*}
L = \sum _{i=1}^{k} - I(Y= i) \log (\hat{{p}}_{i}(X)) \end{eqnarray*}

\begin{eqnarray*}
- I(y \ne i)\log (1 - \hat{{p}}_{i}(X)) \end{eqnarray*}

where *I* is the indicator function. Note that the probabilistic classifier $\hat{{p}}_{i}(X)$ is here defined using the sigmoid function as in Eq. 3, instead of the softmax function. From this follows the classification and rejection procedure:


\begin{eqnarray*}
y=\left\lbrace \begin{array}{@{}l@{\quad }l@{}}\text{reject} & \quad \text{if}\ \hat{{p}}_{i}(X)< t_i,\ \forall i \in \lbrace 1,...,k\rbrace \\ \mathop {\text{argmax}}\limits _{i \in \lbrace 1,..,k\rbrace }\ \hat{{p}}_{i}(X) & \quad \text{otherwise} \end{array}\right. \end{eqnarray*}

For each class $i \in \lbrace 1,...,k\rbrace$, the limit $t_i$ can be calculated as a Gaussian by using the predicted probabilities of the correctly classified points with the mean $\mu _{i}$ as one-half of a Gaussian distribution and constructing the other half by mirroring the points. Using the standard deviation $\sigma$ of this distribution, [33] then obtain the limit:


\begin{eqnarray*}
t_i = max(0.5, 1 - \alpha * \sigma ) \end{eqnarray*}

We adhere to the methodology of the original publication by selecting $\alpha$ as 3. According to [33], this choice functions as an outlier detection measure, as it represents the number of standard deviations a point must be from the mean.

#### Label smoothing

For every example, the model calculates the probability using a softmax function. The loss is then calculated according to [34, 35] as a cross-entropy loss:


\begin{eqnarray*}
L = - \sum _{i=1}^{k} \log \hat{{p}}_{i}(X) q(Y=i) \end{eqnarray*}

Here, $q(Y)$ is the ground-truth distribution. In training, the log-likelihood is maximized for the Dirac delta $q(Y) = \delta _{Y,i}$, which is either 0 when $y \ne i$ or 1. Since fitting to the ground truth can lead to over-likelihood, a simple technique for smoothing the labels is proposed. Using a distribution over labels $\mu (Y)$ and a smoothing parameter $\epsilon$:


\begin{eqnarray*}
q^{\prime }(Y=i) = (1-\epsilon ) \delta _{y,i} + \epsilon \mu (Y=i) \end{eqnarray*}

to replace the Dirac delta $q(Y) = \delta _{Y,i}$. The distribution $\mu (Y)$ is created by using the uniform distribution $\mu (Y) \sim 1/k$ with *k* labels [34, 35].

### Deepdefense architecture and training

#### Matrix and vector scaling—Platt scaling

Matrix and vector scaling are multiclass extensions of Platt scaling. With $\mathbf {z}(X)$ being the logits vector before applying the softmax layer, matrix scaling now applies a linear transformation [31]:


\begin{eqnarray*}
\mathbf {z}^{{scale}}(X)&=&\mathbf {W}\mathbf {z}(X) + \mathbf {b}\\ \mathop {\hat{\mathbf {p}}}(X) &=& \sigma _{SM}(\mathbf {z}^{{scale}}(X)) \end{eqnarray*}

The parameters $\mathbf {W}$ and $\mathbf {b}$ are optimized for the cross-entropy loss on the validation set. Vector scaling uses a vector instead of a matrix [31].

#### Temperature scaling

Temperature scaling is a version of Platt logistic scaling [37] that uses only 1 parameter. It simply uses a parameter *T* to rescale logit scores $\mathbf {z}(X)$ before applying the softmax function.


\begin{eqnarray*}
\mathop {\hat{\mathbf {p}}}(X) = \sigma _{SM}(\mathbf {z}(X)/T) \end{eqnarray*}

An optimal *T* can be received by minimizing the negative log-likelihood on a validation set [31, 37].

### Ensembles

In the context of neural networks, ensembles can be constructed to improve prediction quality or uncertainty estimation. Empirically, using ensembles for uncertainty prediction has already shown promising results, possibly because, unlike stochastic models, they can capture different modes of the underlying solution space [38]. Guo et al. [31] observed that the model capacity negatively influences calibration, which also motivates the choice of ensembles since it allows us to use smaller models and still achieve good accuracy. We build our ensembles by randomly initiating each network’s parameters and predicting the class with the highest confidence in the averaged output of all ensemble members. We choose the lowest confidence that agrees with the determined class assignment as class certainty to get reliable uncertainty estimations.

### General Optimization Strategy

**Figure 1:**
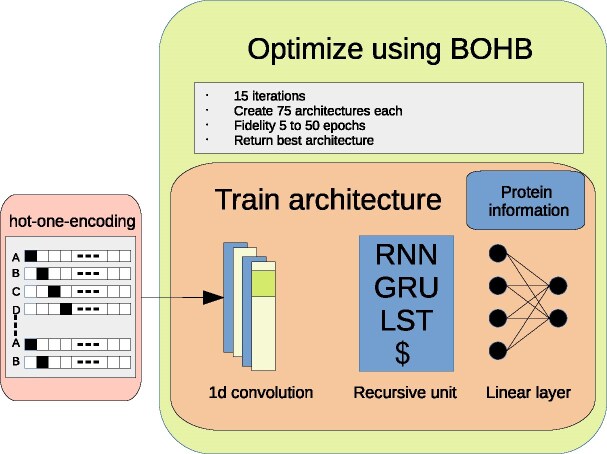
Overview of the training procedure. We separate the data in training, testing, and validation, using 10% of the data for testing and 10% for validation to utilize an early stopping procedure. The proteins are encoded using a one-hot encoding. Additional information can be fed into the linear layers of the used architecture. We optimize this process using BOHB by creating 75 unique architectures in 15 runs for a smaller fidelity. We then tune the resulting best architecture manually on full fidelity. This results in an architecture using a 1D-convolution, which feeds an encoding into a 2-layered bidirectional GRU unit. The output of this unit is then concatenated to prepossessed additional information before the final output is created using a linear classifier. To keep the illustration simple, we show only 3 architectures.

We use Bayesian Optimization with Hyperband (BOHB, see [39]) to optimize 8 baseline architectures on a smaller fidelity. This optimization process affects the kernel sizes, stride, number of channels, dropout, and output dimension of the recursive unit. When using an architecture with a second head, we also optimize the number of nodes used to process the data. The basic structure of this process can be seen in Fig. 1. We additionally optimize a transformer-based architecture for classification, using positional and linear encoding, and optimize the number of heads for the attention layer, dropout, the number of linear layers, and the embedding dimension. The baseline architectures are designed to be relatively shallow to avoid problems with overconfidence in classification [31]. We run 15 iterations for each baseline architecture to create 75 unique, optimized hyperparameter configurations and execute 100 runs. Following the optimization process, we manually improve the best architecture identified by BOHB on the complete fidelity.

**Figure 2:**
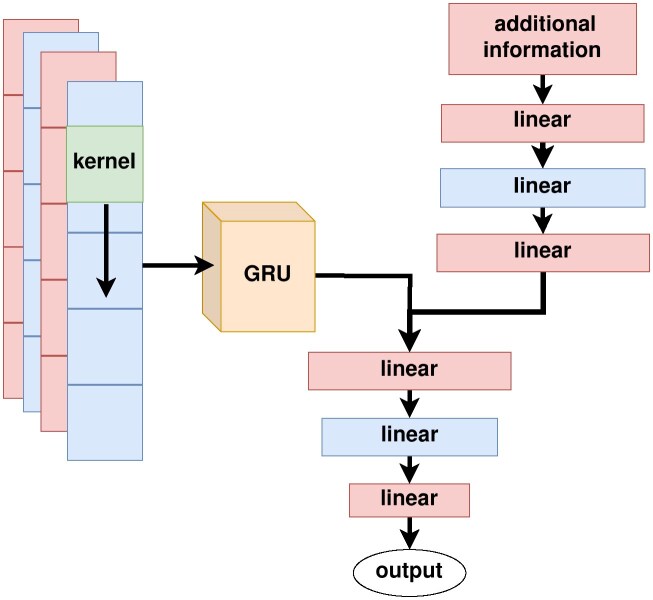
After optimization, the final architecture (with additional information) consists of 1 convolution layer, 2 bidirectional GRU layers, and 3 linear output layers. A second head processes additional information provided by the sequence (see Supplementary Table S1). The outcome of this preprocessing is concatenated with the output of the GRU module.

The final architecture uses 1 convolutional layer with a kernel size of 7 and a stride of 5, followed by 2 bidirectional GRU layers and 3 linear layers to produce the models’ output. The architecture can be seen in Fig. 2. This structure has also previously been shown to create promising results in different classification tasks [30, 40]. The proteins are encoded in a one-hot vector. We use the Adam optimizer and a learning rate of 0.001 and train for 150 epochs. After a third of all epochs have passed, we employ early stopping if the validation loss does not improve for 20 epochs to prevent overfitting. We use a MultiStepLearningRateScheduler to adapt our learning rate by a factor of 0.9 after 3, 12, and then every other 10 epochs up to 100. In this way, we create 3 models we use in an ensemble during testing by predicting the class with the highest average confidence and the probability as the lowest model confidence that agrees with this prediction.

### Cassette pipeline

The pipeline for the classification of cassettes consists of 2 distinct modules. The first module classifies proteins as related to the immune system or unrelated. Proteins found to be related can then be passed on to the second module of the pipeline, which performs a multiclass prediction to annotate the specific immune system.

The results of the multiclass prediction can be grouped into different classes depending on 2 different cutoffs. One cutoff rejects proteins that are considered unrelated to the immune system. The second cutoff determines candidates for a possible later investigation into new immune system types. The resulting classifications are then used to form immune system cassettes according to user specifications.

This design enables us to discern disparities between proteins associated and unassociated with the immune system by evaluating variations in the confidence values predicted by the model. Furthermore, this approach can serve as a valuable tool in distinguishing between proteins already identified as part of the immune system and those that may potentially represent novel additions to our understanding of immune system proteins.

### Prediction quality after optimization

To create a model for our classification scheme, we search over 75 optimized architectures for 8 basic architectures. Figure 1 also shows the optimization process. This search shows a similar distribution for all 8 models (see Supplementary Figs. S2 and S3). The best result overall models were found with the GRU-based model. The validation loss for models with additional information is considerably better than those without additional information. Using this model as a baseline, we optimize it on the whole fidelity. The resulting architecture can be seen in Fig. 2. The transformer-based model probably underperforms here due to some classes having a low number of samples. Training and testing this architecture against immune system–related and immune system–unrelated proteins in a 5-fold cross-validation (CV), we achieve an accuracy of 0.96 and an excellent value for the curve under the receiver operating characteristic (ROC-AUC) of 0.99. Using a maximal sequence homology of 95, we achieve an accuracy of 0.91 and ROC-AUC of 0.96. To test the quality of our architecture, we employ a 10-fold CV and achieve an average accuracy of 0.96 with an average ROC-AUC of 0.995 and weighted area under the precision-recall curve (AUPRC) of 0.979 (results for all classes can be seen in Supplementary Table S2).

Our model undergoes testing across diverse sequence homology levels. At maximal homology (95) between proteins, we find a high accuracy (0.95), ROC-AUC (0.992), and weighted AUPRC (0.973). Maintaining consistently high performance, at a homology of 90, accuracy remains at 0.95 with ROC-AUC (0.994) and weighted AUPRC (0.969). For a homology of 80, we still get a high accuracy (0.93) and good ROC-AUC (0.980) and AUPRC (0.943) values. However, at a homology of 70, accuracy slightly decreases to 0.90 with ROC-AUC (0.978) and AUPRC (0.91). A homology of 60 maintains accuracy at 0.90, ROC-AUC (0.982), and AUPRC (0.90). At a homology of 50, accuracy is 0.83, AUPRC (0.839), and ROC-AUC (0.973), opting for a homology of 60 for DruA and DruB due to sample limitations. A homology of 40, with only 19 classes having sufficient samples, yields an average accuracy of 0.77, ROC-AUC (0.918), and AUPRC (0.755), while the remaining classes are created at a higher homology. In the last run at a homology of 30, only JetA and JetC have enough samples, resulting in an average accuracy of JetA and JetC of 0.5, expected due to the very low number of training samples. All other classes at this cutoff were created at a higher homology.

**Figure 3:**
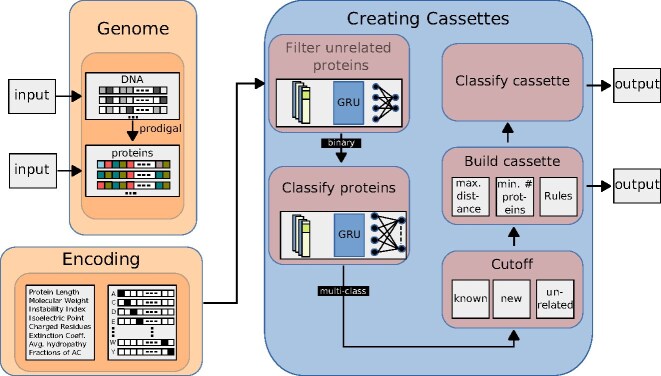
Overview of the classification workflow. The genome is read as nucleotides and translated into proteins using prodigal. Alternatively, proteins can be used as an input. The proteins are encoded in a one-hot fashion, and additional information about the proteins is created. In the first step, these proteins can be filtered using a binary classification model trained on the immune system and unrelated proteins. We use a cutoff that favors the prediction of immune system proteins since using cassettes for classification naturally drives down the false-positive rate. The second classification model now classifies the immune systems and allows us to detect new immune types or reject unrelated proteins. Based on the classification, we build possible cassettes and classify the cassette type by allowing users to choose the maximum distance between proteins and the minimum number of proteins belonging to a rule.

Since our ultimate goal is to predict immune system cassettes, which consist of closely clustered and ordered immune system genes, we get a reduced false-positive rate compared to single-gene classification. Therefore, we can set the cutoff used for the first module at 0.3, which maximizes the accuracy of correctly identified proteins belonging to the immune system. Next, we use our calibrated network and set the first cutoff of the second module as defined in [33] to $\alpha = 3$ and our second cutoff to $\alpha = 4$. The resulting pipeline can also be seen in Fig. 3.

## Results

### Benchmark

Since our method should be able to scan the entire genome and reject unrelated proteins, we explore methods to improve the uncertainty estimations. Details on these methods used for the following benchmark are given in the Materials and Methods section. To create a benchmark, we first train models on the Zorya class for all calibration methods that do not rely on scaling. Then we determine the mean distance of the prediction between the test set and a set of unrelated proteins using 5-fold CV. In the next step, this unrelated class is used to scale the model logits using temperature, matrix, or vector scaling. The result of this benchmark can be seen in Tables 1 and 2. This allows us to test the method’s usefulness directly on the main task to be improved: the rejection of unrelated proteins in a genome-wide search. The highest mean distances were achieved using a combination of scaling (temperature or matrix) with DOC. We did not find a systematical advantage of label smoothing over using softmax in our test bench. For all techniques, we found an improvement in accuracy in using ensembles compared to single networks. Since temperature scaling multiplies the output with a single parameter, it does not change the order between the values for the class prediction, which is not the case for matrix and vector scaling. Here accuracies may drop (see Supplementary Table S3). Due to stable accuracies and an otherwise high mean distance (of 0.30, which is on par with the results achieved by matrix scaling), we choose temperature scaling combined with DOC to scale our methods.

**Table 1:** Benchmark for neural networks in single use

	Softmax		DOC		Smoothing		Penalize		Method dist.		Method acc.	
Scaling	Dist.	Acc.	Dist.	Acc.	Dist.	Acc.	Dist.	Acc.	Mean	SD	Mean	SD
Unscaled	0.10	0.99	0.18	0.99	0.11	0.97	0.12	0.99	0.13	0.0329	0.99	0.0079
Temp scaling	0.12	0.99	0.20	0.99	0.14	0.97	0.14	0.99	0.15	0.0306	0.99	0.0079
Vector scaling	0.15	0.80	−0.14	0.55	0.21	0.96	0.10	0.75	0.08	0.1321	0.77	0.1461
Matrix scaling	0.15	0.55	0.02	0.63	0.17	0.39	0.14	0.64	0.12	0.0562	0.55	0.0984
**Scaling mean**	0.13	0.83	0.07	0.79	0.16	0.83	0.12	0.84				
**Scaling SD**	0.022	0.179	0.138	0.202	0.036	0.251	0.015	0.155				

**Table 2:** Benchmark for neural networks in ensemble use

	Softmax		DOC		Smoothing		Penalize		Method dist.		Method acc.	
Scaling	Dist.	Acc.	Dist.	Acc.	Dist.	Acc.	Dist.	Acc.	Mean	SD	Mean	SD
Unscaled	0.10	0.99	0.29	1.0	0.11	0.99	0.11	1.0	0.15	0.0777	1.0	0.0045
Temp scaling	0.13	0.99	**0.30**	1.0	0.14	0.99	0.14	1.0	0.18	0.0691	1.0	0.0045
Vector scaling	0.10	0.97	0.15	0.64	0.14	0.76	0.12	0.78	0.13	0.0184	0.79	0.1202
Matrix scaling	0.13	0.58	**0.30**	0.74	0.11	0.78	0.18	0.87	0.18	0.0757	0.74	0.1053
**Scaling mean**	0.12	0.88	0.26	0.84	0.12	0.88	0.14	0.91				
**Scaling SD**	0.014	0.175	0.064	0.161	0.014	0.109	0.026	0.092				

The tables show the average distance between related and unrelated proteins for a combination of methods relying on scaling or modifying the training process. Results are given for both single (using only 1 neural network, Table 1) and ensemble (using the output of 3 neural networks, Table 2) use. We see the best results for the DOC method in ensemble use in combination with temperature and matrix scaling (see bolded values). Here, we observe an average difference of 30% in classification probabilities between proteins unrelated to the immune system and those related to the immune system, which allows us to clearly distinguish the 2 different classes and will be used in the subsequent analysis. We furthermore noted that the use of an ensemble outperforms the single use in most cases when measuring the distance and, in all cases, when determining the accuracy. We additionally assess the overall effectiveness of the scaling approach and calibration method employed. Our findings reveal that temperature scaling exerts the biggest influence, without adversely affecting the overall accuracy. For ensemble use, DOC showed the best overall performance, displaying moderate variation over different scaling methods, as indicated by the corresponding standard deviation.

### Detecting cassettes in bacteria, archea, plasmids, and phage

**Figure 4:**
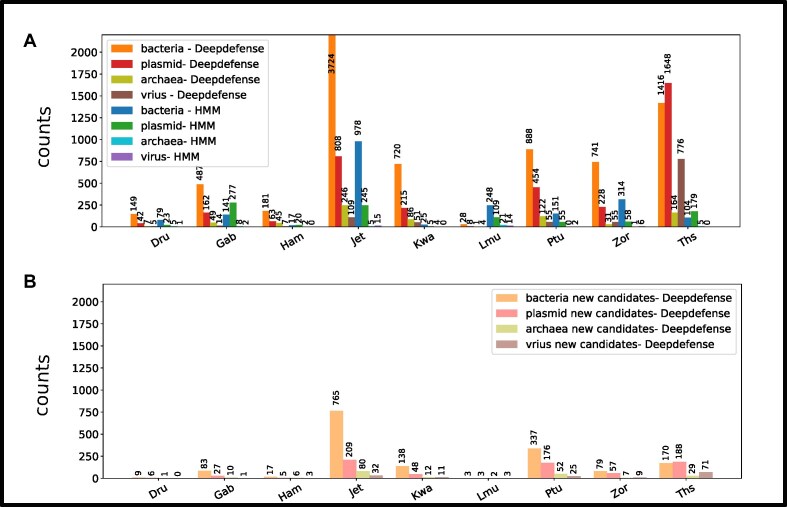
Distribution of immune systems detected using our method and HMM homolog search. Despite the smaller dataset, more immune system candidates were found in bacteria genomes than in plasmids. The lower number of systems belonging to archaea is partially due to the much smaller dataset. Additionally, we did find some cassettes in a bigger phage dataset. The x-axis shows the immune system type, while the y-axis shows the counts of the found immune system cassettes. The barplot (B) shows the number of candidates discovered for further investigation by Deepdefense.

Forming cassettes of related proteins in the genome exploits the property of immune system proteins to cluster close to each other in so-called defense islands. We choose a maximal distance of 2 genes between classes belonging to a cassette rule to detect possible cassettes. Since the immune system Shedu is not known to form a cassette, we exclude it from the search. This distance considers the differences in the location of genes belonging to a system. It allows for some variability while using the cluster forming of immune systems. We set the minimum number of genes belonging to a system for identification to 2 since many cassettes only have up to 2 belonging candidates. These parameters can be set customarily. We run our algorithm against a database of 18,441 plasmids, 799 archaea, 7,308 bacteria, and a phage dataset of 2,689 samples. The search yielded 3,765 candidates in plasmids, with 562 candidates belonging to a potentially new type. For bacteria, we find 8,751 candidates and 1,608 potentially new immune system cassettes, with a ratio of 0.18. We find a higher ratio of 0.25 in archaea, with 771 known and 195 potentially new types. Within the phage dataset, we find 1,078 known types, which mostly belonged to the Thoeris type. The resulting distribution for all discovered cassette types for our Deepdefense (RRID: SCR_025346) approach and the results for an HMM-based (HMM-TM RRID: SCR_006816) search can be seen in Fig. 4A. Both distributions show a spike for Wadjet-based and Thoeris-based cassettes, while fewer immune system cassettes were found in plasmids, despite the larger dataset (probably due to the smaller genome size). The HMM and our Deepdefense method discover some cassettes in virus genomes. This might give a hint to the potential origin of the immune system. We also discover potential candidates for currently unknown immune system proteins using our second cutoff. Figure 4B shows the relatively low number of potential candidates discovered by Deepdefense. We find a small spike for the Wadjet immune system again and, surprisingly, for Septu.

**Figure 5:**
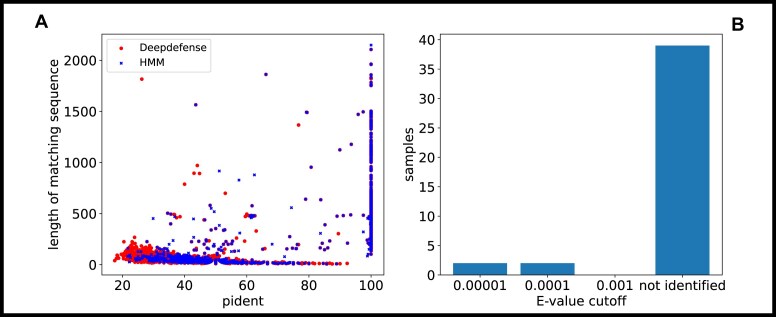
Presented is a scatterplot (A) illustrating the relationship between pident (percentage of identical positions) and the length of matching sequences for all proteins within a cassette identified by both Deepdefense (10,335 samples) and a hidden Markov model (2,348 samples) in bacterial genomes. It is noteworthy that there is significant overlap between the 2 methods; however, Deepdefense excels in detecting proteins with a lower sequence similarity. The values for pident and the length of matching sequences were generated using BLAST, which mapped annotated proteins against the original dataset used for training purposes. The barplot on the right (B) depicts the distribution of E-values for sequences with moderate pident (>40, <50) and matching sequence length (>30), where we also identify neighboring proteins associated with immune system cassettes. Despite significant sequence homology and appropriate neighborhood alignment, a notable number of proteins remain unidentified by the existing HMM-based approach.

To conduct a comparison between proteins identified by DeepDefense and those identified through an HMM-based approach, we align the predicted proteins with the training dataset and analyze the percentage identity (pident) and the length of homologous subsequences, as depicted in Fig. 5. The resulting scatterplot reveals a substantial and anticipated overlap among highly homologous sequences. Notably, DeepDefense predicts a larger set of sequences related to the immune system across a spectrum of homology levels, including both highly homologous and less homologous sequences. This is particularly noticeable when discerning sequences with lower homology levels. Furthermore, we employ sequences featuring neighboring proteins identified as affiliated with immune system cassettes and examine the E-value distribution for sequences exhibiting reasonably high homology and matching sequence length in Fig. 5. Despite substantial sequence homology and suitable neighborhood alignment, a considerable number of proteins remain unidentified by an HMM-based approach.

Additionally, we conducted a comparative analysis of the outputs generated by the HMM-based approach PADLOC [27, 41] and Deepdefense, focusing on 76 genomes sourced from the PADLOC website. Deepdefense identified a total of 885 proteins associated with defense systems, whereas PADLOC’s prediction encompassed 199 proteins affiliated with the Doron systems. Notably, an intersection of 55 proteins was observed, underscoring the distinct cassette analysis approaches employed by both methods. Among the pool of 144 prospective candidates for novel immune systems, PADLOC classified a mere 5 as systems unrelated to the Doron framework.

### Distribution of detected immune systems in 3 phyla

**Figure 6:**
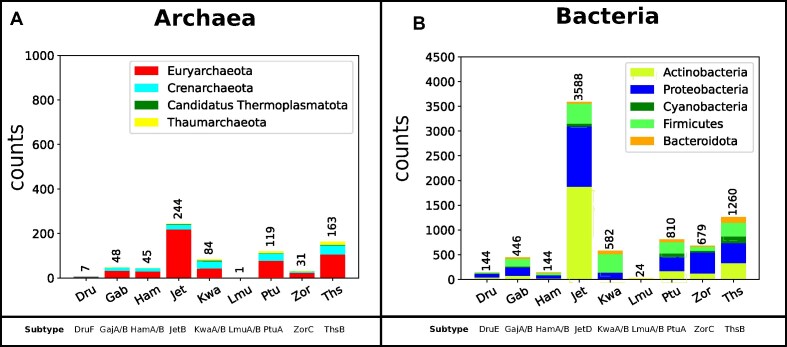
Immune system class distribution for archaea (A) and bacteria (B), color-coded for available phyla. The y-axis shows the counts of the immune cassettes, and the x-axis shows the immune system types. In bacteria, we find a majority of detected cassettes belonging to Wadjet, with 2 phyla particularly prominent. Within archaea, we mostly find cassettes for 1 phylum.

**Figure 7:**
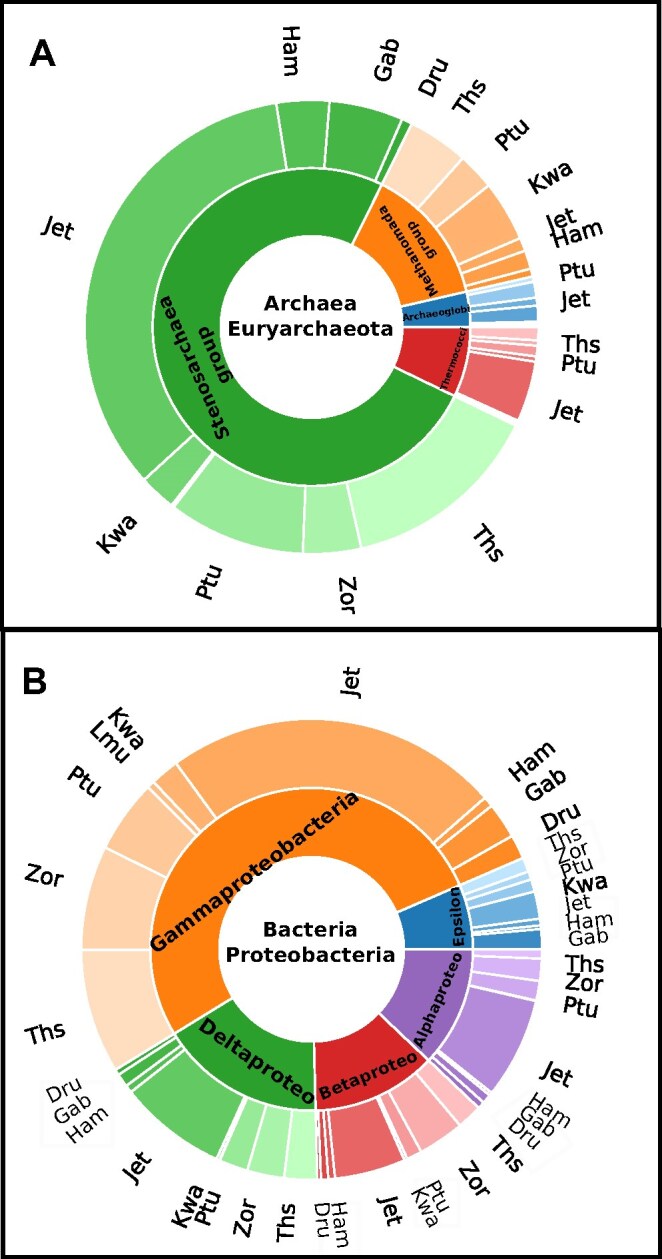
The distribution of detected cassette classes in 3 phyla for multiple classes. Phyla Euryarchaeota (A), with classes Stenosarcaea group, Archeaoglobi, Thermococci, and Methanomado group, and Proteobacteria (B), with classes Gammaproteobacteria, Deltaproteobacteria, Betaproteobacteria, Alphaproteobacteria, and Epsilonproteobacteria. The outer circle then shows the distribution of the immune system cassettes.

Using the distribution of phyla for all immune systems (as seen in Fig. 6), we choose the highest expressed phyla and plot the distribution in Fig. 7. A major phylum of Archaea was Euryarchaeota, with the majority classes Stenosarcaea, Archeaoglobi, Thermococci, and Methanomado and a total number of 535 elements. In total, 399 immune cassettes were identified in Stenosarchaea, 19 in Archaeoglobi, 38 in Thermococci, and 75 in Methanomado. For bacteria, we identify 2 major phyla, Actinobacteria (with a total number of 2,642 cassettes, see Supplementary Fig. S5) and Proteobacteria (with a total number of 2,785 cassettes). The former consists mostly of the classes Bifidobacteriales (265 cassettes), Micrococcales (214 cassettes), Streptomycetales (791 cassettes), and Corynebacteriales (585 cassettes), and the latter consists of the classes Gammaproteobacteria (1,449 cassettes), Deltaproteobacteria (335 cassettes), Betaproteobacteria (461 cassettes), Alphaproteobacteria (347 cassettes), and Epsilonproteobacteria (180 cassettes).

## Conclusion

We developed a method for automatically annotating prokaryote immune systems using deep learning. Here, we had to achieve the following 2 goals for accurately detecting cassettes of immune systems in prokaryotes: (i) to create models that can classify already known types of immune systems and (ii) to create a mechanism that allows for the rejection of unrelated proteins. We achieved the first goal using a BOHB-based optimization process over some baseline deep neural architectures, creating an optimized model that produces good classification accuracies in this complex domain. The second goal is achieved in 2 ways. First, we create a model rejecting proteins dissimilar to proteins known to belong to the immune system. Second, we calibrate our model for classifying immune system proteins to get a reliable confidence measurement for our predictions, which allows us to reject unrelated proteins. For this purpose, we first create a test bench that compares the effect of combining different methods on the mean distance between related and unrelated proteins. This distance is maximized using a combination of DOC and temperature scaling. Using the created calibrated model, we can also determine potential candidates for new immune system subtypes using a second cutoff. A clustering mechanism then matches the spatially close immune systems to a cassette. This allows us to scan the genome of archaea, bacteria, and plasmids for immune systems. Given this set of potential new candidate proteins, the obvious next step is to investigate these candidates that potentially belong to a new subtype of the immune system experimentally.

## Supplementary Material

giae062_GIGA-D-23-00300_Original_Submission

giae062_GIGA-D-23-00300_Revision_1

giae062_GIGA-D-23-00300_Revision_2

giae062_GIGA-D-23-00300_Revision_3

giae062_Response_to_Reviewer_Comments_Original_Submission

giae062_Response_to_Reviewer_Comments_Revision_1

giae062_Response_to_Reviewer_Comments_Revision_2

giae062_Reviewer_1_Report_Original_Submission Hao Lin -- 11/21/2023

giae062_Reviewer_1_Report_Revision_1 Hao Lin -- 3/6/2024

giae062_Reviewer_2_Report_Original_Submission Jiawei Wang -- 11/22/2023

giae062_Reviewer_2_Report_Revision_1 Jiawei Wang -- 2/27/2024

## Data Availability

The data used for training are publicly available as part of the publication [26]. The used HMM models are part of the publication [27]. Additionally, data were taken from the PADLOC website [41]. The trained model, as well as the additionally created training data, can be found in our GitHub repository. Snapshots of our code and other data further supporting this work are openly available in the *GigaScience* repository, GigaDB [42].

## Additional Files

**Supplementary Table S1**. The table shows the additional protein information computed for the second model input.

**Supplementary Table S2**. The table shows the class accuracies using the multiclass prediction model based on GRU using additional information executed on 10-fold CV.

**Supplementary Table S3**. The table shows the effect of the chosen combination of methods on the accuracy of the model. In our test bench, we find high accuracies for unscaled and temperature-scaled methods over all possible combinations and lower accuracies for matrix and vector scaling. This can be explained due to the outcome invariant scaling that temperature scaling uses.

**Supplementary Fig. S1**. A more detailed description of the final architecture used for classification.

**Supplementary Fig. S2**. Distribution of optimization results using BOHB without additional information. We optimize over the kernel sizes, stride, number of channels, dropout, and output dimension of the recursive unit and over the number of heads for the attention layer, dropout, the number of linear layers, and embedding dimension when using the transformer-based classification model. We can see a slightly higher mean for the GRU-based models. The best architecture here also belongs to GRU models (see outlier).

**Supplementary Fig. S3**. In addition to optimization without additional information, we optimize over the number of nodes used to process the additional data. The validation loss is lower for all baseline architectures, using additional information compared to the version without additional information. Again, GRU-based models do perform best.

**Supplementary Fig. S4**. Overview over distribution for phyla Archaea Eurayarchaeota (A) and Bacteria Proteobacteria with associated classes. The outer circle also shows the distribution of the cassettes found in each class.

**Supplementary Fig. S5**. Overview over distribution for phyla Archaea Eurayarchaeota (A) and Bacteria Achinobacteria with associated classes. The outer circle also shows the distribution of the cassettes found in each class.

## Abbreviations

BLAST: Basic Local Alignment Search Tool; BOHB: Bayesian Optimization with Hyperband; CRISPR: clustered regularly interspaced short palindromic repeats; DOC: Deep Open Classification; HMM: hidden Markov model; ROC-AUC: curve under the receiver operating characteristic; PRAUC: area under the precision-recall curve; ML: machine learning.

## Availability of Source Code and Requirements

- Project name: Deepdefense
- Project homepage: https://github.com/SvenHauns/Deepdefense
- Zenodo DOI: https://doi.org/10.5524/102550
- Operating system: Platform independent
- Programming language: Python
- Other requirements: see public environment file
- Publicly available under MIT license
- Registered as Deepdefense in bio.tools database
- RRID: SCR_025346
