# Supplementary material for: Deepdefense: annotation of immune systems in prokaryotes using deep learning
Source: Gigascience. 2024 Oct 10;13:giae062. doi: 10.1093/gigascience/giae062 (PMC11959188; doi:10.1093/gigascience/giae062)
Supplement: giae062_GIGA-D-23-00300_Original_Submission [file giae062_giga-d-23-00300_original_submission.pdf]

GigaScience

Deepdefense: Annotation of immune systems in prokaryotes using DeepLearning

--Manuscript Draft--

**Manuscript Number:**	GIGA-D-23-00300
**Full Title:**	Deepdefense: Annotation of immune systems in prokaryotes using DeepLearning
**Article Type:**	Technical Note
**Funding Information:**	Deutsche Forschungsgemeinschaft (BA 2168/23-1/) — Professor Omer Alkhnbashi<br>Deutsche Forschungsgemeinschaft (BA 2168/25-1) — Herr Sven Hauns
**Abstract:**	Background Due to a constant evolutionary arms race, archaea and bacteria have evolved an abundance and diversity of immune responses to protect themselves against phages. Since the discovery and application of CRISPR-Cas adaptive immune systems, numerous novel candidates for immune systems have been identified. Previous approaches to identifying these new immune systems rely on HMM-based homolog searches or use labor-intensive and costly wet-lab experiments. To aid in finding and classifying immune systems, we use machine learning to classify already known immune system proteins and discover potential candidates in the genome. Neural networks have shown promising results in classifying and predicting protein functionality in recent years. However, these approaches frequently rely on the assumption of a closed world, which is often different in practice.<br>Results In this work, we explore neural networks for immune protein classification and deal with different methods for rejecting unrelated proteins in a genome-wide search, and establish a benchmark. Then, we optimize our approach for accuracy. Based on this, we develop an algorithm called Deepdefense to predict immune cassette classes based on a genome. Finally, we test our approach for detecting immune systems in the genome against an HMM-based method.<br>Conclusions Deepdefense can automatically detect genes and define cassette annotations and classifications using two model classifications. This is achieved by creating an optimized deep learning model to annotate immune systems, in combination with calibration methods, and a second model to enable the scanning of an entire genome.
**Corresponding Author:**	Sven Hauns<br>Albert-Ludwigs-Universität Freiburg: Albert-Ludwigs-Universitat Freiburg<br>Freiburg, GERMANY
**Corresponding Author Secondary Information:**	
**Corresponding Author's Institution:**	Albert-Ludwigs-Universität Freiburg: Albert-Ludwigs-Universitat Freiburg
**Corresponding Author's Secondary Institution:**	
**First Author:**	Sven Hauns
**First Author Secondary Information:**	
**Order of Authors:**	Sven Hauns
	Omer Alkhnbashi
	Rolf Backofen
**Order of Authors Secondary Information:**	
**Additional Information:**	

Powered by Editorial Manager® and ProduXion Manager® from Aries Systems Corporation

Question	Response
Are you submitting this manuscript to a special series or article collection?	No
**Experimental design and statistics**<br><br>Full details of the experimental design and statistical methods used should be given in the Methods section, as detailed in our Minimum Standards Reporting Checklist. Information essential to interpreting the data presented should be made available in the figure legends.<br><br>Have you included all the information requested in your manuscript?	Yes
**Resources**<br><br>A description of all resources used, including antibodies, cell lines, animals and software tools, with enough information to allow them to be uniquely identified, should be included in the Methods section. Authors are strongly encouraged to cite Research Resource Identifiers (RRIDs) for antibodies, model organisms and tools, where possible.<br><br>Have you included the information requested as detailed in our Minimum Standards Reporting Checklist?	Yes
**Availability of data and materials**<br><br>All datasets and code on which the conclusions of the paper rely must be either included in your submission or deposited in publicly available repositories (where available and ethically appropriate), referencing such data using a unique identifier in the references and in the "Availability of Data and Materials" section of your manuscript.	Yes

Have you have met the above requirement as detailed in our Minimum Standards Reporting Checklist?	

main manuscript as pdf Click here to access/download;Manuscript;Deepdefense.pdf

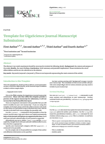

GigaScience, 2023, 1–9

doi: xx.xxxx/xxxx
Manuscript in Preparation
Paper

PAPER

Deepdefense: Annotation of immune systems in prokaryotes using DeepLearning

Sven Hauns[1], Omer S. Alkhnbashi[2,3,*] and Rolf Backofen[1,4,*]

[1]Bioinformatics group,Department of Computer Science, University of Freiburg,Georges-Köhler-Allee 101,79110,Germany and [2]Information and Computer Science department King Fahd University of Petroleum & Minerals, Dhahran, 31261, Saudi Arabia and [3]Interdisciplinary Research Center for Intelligent Secure Systems (IRC-ISS), King Fahd University of Petroleum & Minerals, Dhahran, 31261, Saudi Arabia and [4]Signalling Research Centres BIOSS and CIBSS, University of Freiburg, Schänzlestr. 18, 79104 Freiburg, Germany

[*]Corresponding authors: omer.khnbashi@kfupm.edu.sa backofen@informatik.uni-freiburg.de

Abstract

Background Due to a constant evolutionary arms race, archaea and bacteria have evolved an abundance and diversity of immune responses to protect themselves against phages. Since the discovery and application of CRISPR-Cas adaptive immune systems, numerous novel candidates for immune systems have been identified. Previous approaches to identifying these new immune systems rely on HMM-based homolog searches or use labor-intensive and costly wet-lab experiments. To aid in finding and classifying immune systems, we use machine learning to classify already known immune system proteins and discover potential candidates in the genome. Neural networks have shown promising results in classifying and predicting protein functionality in recent years. However, these approaches frequently rely on the assumption of a closed world, which is often different in practice.
Results In this work, we explore neural networks for immune protein classification and deal with different methods for rejecting unrelated proteins in a genome-wide search, and establish a benchmark. Then, we optimize our approach for accuracy. Based on this, we develop an algorithm called Deepdefense to predict immune cassette classes based on a genome. Finally, we test our approach for detecting immune systems in the genome against an HMM-based method.
Conclusions Deepdefense can automatically detect genes and define cassette annotations and classifications using two model classifications. This is achieved by creating an optimized deep learning model to annotate immune systems, in combination with calibration methods, and a second model to enable the scanning of an entire genome

Key words: immune systems; deep learning; classification; genome

Introduction

Viruses are the most abundant biological entities in biospheres such as soil and sea, vastly outnumbering prokaryotes [1, 2, 3, 4]. Given such an abundance of numbers, phages, and plasmids frequently attack bacteria and archaea. Because of such attacks and the resulting arms race, they have evolved various techniques to defend themselves [5, 6].

These techniques can be grouped into two general defense mechanisms based on the principle of their action: 1) innate or adaptive immunity, and 2) programmed cell death and dormancy [7, 8]. Innate immunity is often based on restriction-modification (R-M) systems, where a restriction enzyme cuts unmethylated DNA. As the own DNA is methylated, this protects any unmethylated foreign DNA [6]. In contrast, the CRISPR-Cas systems provide adaptive immunity, which recognizes and degrades specific viral nucleic acids [9]. The ability of the CRISPR systems to precisely cut and integrate viral nucleic acids into the bacteria genome has led to the creation of important new genetic tools, such as Cas9 for gene editing [10, 11, 12]).

The success of CRISPR/Cas9 has fuelled the search for new members of the CRISPR-Cas systems and led to many different computational approaches, both scoring or machine learn-

Compiled on: October 4, 2023.
Draft manuscript prepared by the author.

Key Points

- We develop a deep learning based approach for the classification of Doron immune systems in whole genomes based on two classifiers.
- The first classifier filters proteins most likely not belonging to the immune system, while the second classifier classifies immune system sub-classes or rejects unrelated data.
- The resulting output classification is then used to build specific cassettes for each immune system type.
- We improve the model calibration using different methods to reject unrelated data reliably.
- Using the calibrated model, we also suggest candidates which may constitute a new immune system.

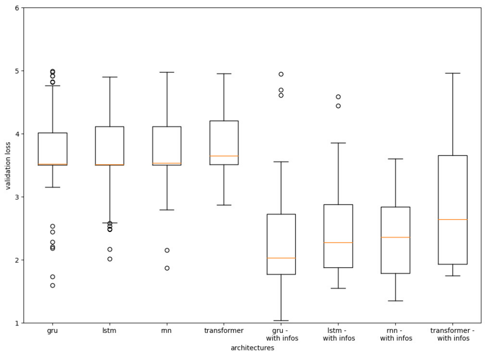

Figure 1. Distribution of optimization results using BOHB without additional information. We optimize over the kernel sizes, stride, number of channels, dropout, and output dimension of the recursive unit and the number of heads for the attention layer, dropout, the number of linear layers, and embedding dimension when using the transformer-based classification model. We can see a slightly higher mean for the GRU-based models. The best architecture here also belongs to GRU models (see outlier). Additionally to optimization without additional information, we also optimize over the number of nodes used to process the additional data. The validation loss is lower for all baseline architectures, using additional information compared to the version without additional information. Again GRU based models do perform best.

ing (ML) based, to identify new members of the CRISPR-Cas system [13, 14, 15, 16, 17, 18, 19, 20, 21, 22, 23]. However, no ML approach exists to date to identify immune systems different from CRISPR, and to suggest candidates for further exploration.

To set up such an ML approach for non-CRISPR immune systems, we have to ask ourselves whether there is some chance to detect an immune system in some prokaryotic genome. The estimated proportion of genes coding for immune systems varies from about 0.0001% to 10% [24]. The given distributions for each defense system show the lower limit because many more variants need to be identified due to the rapid mutation of defense genes. The number of defense genes in a genome shows a linear scaling with the size of the genome [8]. The genomes responsible for immune response are often close together and form so-called defense islands [25]. In the following, we would like to exploit this property to predict immune cassettes based on gene predictions ultimately. Our approach focuses on identifying immune systems identified in [26], named Druantia, Gabija, Hachiman, Kiwa, Lamassu, Septu, Shedu, Thoeris, Wadjet, and Zorya. Following [27], these systems are called the 'Doron systems.'

In this work, we present a deep learning approach called Deepdefense, designed to capture much of the relevant information used in manual annotation. It is purely based on features for the different protein sequences related to immune systems. The proposed approach solves the problem of annotating and classifying Doron defense gene systems into types and subtypes. As our features for the deep learning approach correspond to evidence for immune-related protein sequences, we can determine proteins whose evidence is critical for predicting types and subtypes. Since we want to use our tool to scan the entire genome, we need to be able to reject unrelated proteins reliably. We try to improve the problems caused by this closed-world assumption [28] by using techniques designed to improve model calibration. Furthermore, we compare this approach with the recently published approach based on the HMM (hidden markov model) homology search [27] and demonstrate that Deepdefense can identify more immune systems in prokaryotic genomes. Additionally, the tool identifies potential new immune systems related to the Doron systems.

Data Description

Our dataset consists of 21196 unique validated samples [26]. We separate our dataset in a stratified training, test, and validation split, using a 10-fold CV split to ensure enough training data samples are available for subclasses with few samples. Another 10 % of the remaining dataset is used as a validation set for early stopping. This results in 17169 data samples for training, 1908 for validation, and 2119 for testing in each CV split. We also create test sets using 10 % of the data with a maximal sequence homology of 95, 90, and 80, respectively.

The dataset we used was imbalanced for types, consisting of 1263 Durantia samples, 3723 Gajiba samples,1529 Hachiman samples, 6882 Wadjet samples, 637 Lamassu samples, 2807 Septu samples, 647 Shedu samples, 1097 Thoeris samples, 745 Kiwa samples, 1866 Zorya samples. Hence there were only roughly one-tenth of the samples of Lamassu than for Wadjet. We weight the samples during training according to their distribution in the dataset to prevent overfitting to majority classes [29], by dividing the total number of samples by the number of samples in a class multiplied by the number of classes. Additionally, we use a dataset with 21196 samples from bacteria and archaea that have an unrelated function to the immune system, also ensuring a low similarity to known defense system by sequence homology and HMM based search. In addition to cross-validation, we also use 10 % of the data for an independent test dataset with a maximal sequence homoglogy of 95. We create additional information characterizing the used proteins as in [30].

Results

Prediction Quality after Optimization

To create a model for our classification scheme, we search over 75 optimized architectures for eight basic architectures. The details of the optimization procedure and architectures can be found in the method section. This search shows a similar distribution for all eight models. The best result overall models were found with the GRU-based model. The validation loss for models with additional information is considerably better than those without additional information. Using this model as a baseline, we optimize it on the whole fidelity. The resulting architecture can be seen in

Table 1. Benchmark for neural networks in single use

scaling	softmax	DOC[1]	smoothing	punish	**avg-dist**	**avg-accuracy**
unscaled	0.10	0.18	0.11	0.12	0.13	0.986
temp scaling	0.12	0.20	0.14	0.14	0.15	0.986
vector scaling	0.15	-0.13	0.21	0.10	0.08	0.77
matrix scaling	0.15	0.02	0.17	0.14	0.12	0.55
**avg-dist**	0.13	0.16	0.06	0.12		
**avg-accuracy**	0.83	0.79	0.83	0.84		

Table 2. Benchmark for neural networks in ensemble use

scaling	softmax	DOC[1]	smoothing	punish	**avg-dist**	**avg-accuracy**
unscaled	0.11	0.29	0.11	0.11	0.15	0.995
temp scaling	0.13	**0.30**	0.14	0.14	0.18	0.995
vector scaling	0.10	0.15	0.14	0.12	0.13	0.78
matrix scaling	**0.30**	0.11	0.18	0.18	0.7	
**avg-dist**	0.12	0.26	0.12	0.14		
**avg-accuracy**	0.88	0.84	0.88	0.91		

The tables show the average distance between related and unrelated proteins for a combination of methods relying on scaling or modifying the training process. Results are given for both single (using only one neural network, table 1) and ensemble (using the output of three neural networks, table 2) use. We see the best results for the DOC method in combination with temperature and matrix scaling. The use of an ensemble outperforms the single use in most cases when measuring the distance and, in all cases, when determining the accuracy.

[1]DOC: Deep Open Classification.

Fig. 4. The transformer-based model probably underperforms here due to some classes having a low number of samples. Training and testing this architecture against immune-system-related and immune-system-unrelated proteins in a 5-fold CV, we achieve an accuracy of 0.96 and an excellent ROC-AUC of 0.99. Using a maximal sequence homology of 95 we achieve an accuracy of 0.91 and ROC-AUC of 0.96. To test the quality of our architecture, we employ a 10-fold CV and achieve an average accuracy of 0.96 with an average ROC-AUC of 0.99 and and weighted AUPRC of 0.98 (results for all classes can be seen in the supplementary Table S2). We also test our model, ensuring a maximal sequence homology of 95 between training and test set and achieving an accuracy of of 0.96, ROC-AUC of 0.985, and weighted AUPRC of 0.965. With a maximal sequence homology of 90, we achieve an accuracy of 0.95, ROC-AUC of 0.99, and weighted AUPRC of 0.96. Finally, a test set using a maximal sequence homology of 85 achieves an accuracy of 0.94, ROC-AUC of 0.99, and weighted AUPRC of 0.96. Since our ultimate goal is to predict immune system cassettes, which consist of closely clustered and ordered immune system genes, we get a reduced false-positive rate compared to single-gene classification. Therefore we can set the cutoff used for the first module at 0.3, which maximizes the accuracy of correctly identified proteins belonging to the immune system. Next, we use our calibrated network and set the first cutoff of the second module as defined in [31] to $\alpha = 3$, and our second cutoff to $\alpha = 4$. The resulting pipeline can also be seen in Fig. 5.

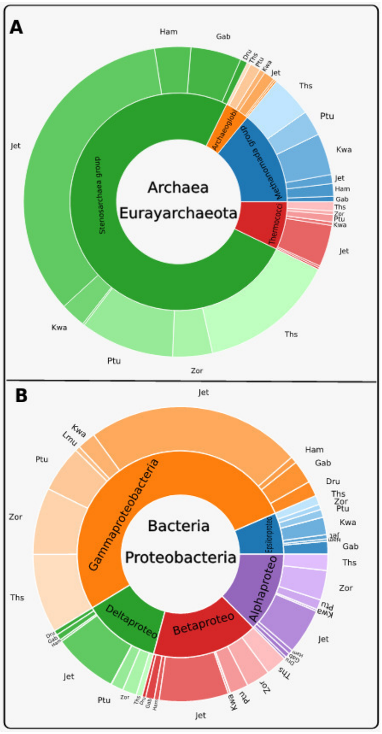

Figure 2. The distribution of detected cassette classes in 3 phyla for multiple classes. Phyla Euryarchaeota (A) with classes Stenosarcaea group, Archeaoglobi, Thermococci, and Methanomado group, and Proteobacteria (B) with classes Gammaproteobacteria, Deltaproteobacteria, Betaproteobacteria, Alphaproteobacteria, Epsilonproteobacteria. The outer circle then shows the distribution of the immune system cassettes.

Benchmark

Since our method should be able to scan the entire genome and reject unrelated proteins, we explore methods to improve the uncertainty estimations. Details on these methods used for the following benchmark are given in the method section. To create a benchmark we first train models on the Zorya class for all calibration methods that do not rely on scaling. Then we determine the mean distance of the prediction between the test set and a set of unrelated proteins using 5-fold CV. In the next step, this unrelated class is used to scale the model logits using temperature, matrix, or vector scaling. The result of this benchmark can be seen in table 1) and 2). This allows us to test the method's usefulness directly on the main task to be improved: the rejection of unrelated proteins in a genome-wide search. The highest mean distances were achieved using a combination of scaling (temperature or matrix) with DOC (Deep Open Classification). We did not find a systematical advantage of label smoothing over using softmax in our test bench. For all techniques, we found an improvement in accuracy in using ensembles compared to single networks. Additionally, we find a moderate improvement in the mean distance of the prediction between the test

set and the set of unrelated proteins (0.1 for DOC and temperature scaling). Since temperature scaling multiplies the output with a single parameter, it does not change the order between the values for the class prediction, which is not the case for matrix and vector scaling.

Here accuracies may drop (see supplementary Table S1). Due to stable accuracies and an otherwise high mean distance (of 0.30, which is on par with the results achieved by matrix scaling), we choose temperature scaling combined with DOC to scale our methods.

Detecting cassettes in bacteria, archea and plasmids and phage

Forming cassettes of related proteins in the genome exploits the property of immune system proteins to cluster close to each other in so-called defense islands. We choose a maximal distance of two genes between classes belonging to a cassette rule to detect possible cassettes. Since the immune system Shedu is not known to form a cassette, we exclude it from the search. This distance considers the differences in the location of genes belonging to a system. It allows for some variability while using the cluster forming of immune systems. We set the minimum number of genes belonging to a system for identification to two since many cassettes only have up to two belonging candidates. These parameters can be set customarily. We run our algorithm against a database of 18441 plasmids, 799 archaea, 7680 bacteria, and a phage dataset of 2689 samples. The search yielded 3628 candidates in plasmids, with 531 candidates belonging to a potentially new type. For bacteria, we find 8334 candidates and 1551 potentially new immune system cassettes, with a ratio of 0.19. We find a higher ratio of 0.26 in archaea, with 751 known and 193 potentially new types. Within the phage dataset, we find 1076 known types, which mostly (72 %) belonged to the Thoeris type. The resulting distribution for all discovered cassette types for our Deepdefense approach and the results for an HMM-based search can be seen in Fig.˜6a. Both distributions show a spike for Wadjet-based and Thoeris-based cassettes, while fewer immune system cassettes were found in plasmids, despite the larger dataset (probably due to the smaller genome size). The HMM and our Deepdefense method discover some cassettes in Virus genomes. This might give a hint to the potential origin of the immune system. We also discover potential candidates for currently unknown immune system proteins using our second cutoff. Fig.˜6b shows the relatively low number of potential candidates discovered by Deepdefense. We find a small spike for the Wadjet immune system again and, surprisingly, for Septu. Furthermore, we conducted a comparative analysis of the outputs generated by the HMM-based approach PADLOC [27, 32] and Deepdefense, focusing on 69 genomes sourced from the PADLOC website. Deepdefense identified a total of 885 proteins associated with defense systems, whereas PADLOC's prediction encompassed 177 proteins affiliated with the Doron systems. Notably, an intersection of 55 proteins was observed, underscoring the distinct cassette analysis approaches employed by both methods. Among the pool of 144 prospective candidates for novel immune systems, PADLOC classified a mere five as systems unrelated to the Doron framework.

Distribution of detected immune systems in three phyla

Using the distribution of phyla for all immune systems (as seen in Fig. 7), we choose the highest expressed phyla and plot the distribution in Fig.˜2. A major phylum of Archaea was Euryarchaeota, with the classes Stenosarcaea, Archeaoglobi, Thermococci, and Methanomado and a cumulative sum of 531 elements. Three hundred ninety-nine immune cassettes were identified in Stenosarcaea, 19 in Archeaoglobi, 38 in Thermococci, and 75 in Methanomado. For bacteria, we identify two major phyla, Actinobacteria (with a total number of 533 cassettes, see supplementary Figure S6) and Proteobacteria (with a total number of 2768 cassettes). The former consists of the classes Bifidobacteriales (194 cassettes), Micrococcales (54 cassettes), Streptomycetales(140 cassettes), and Corynebacteriales (145 cassettes), the latter of the classes Gammaproteobacteria (1445 cassettes), Deltaproteobacteria (335 cassettes), Betaproteobacteria (461 cassettes), Alphaproteobacteria (347 cassettes), Epsilonproteobacteria (180 cassettes). Utilizing our second cutoff, we search our dataset for new immune system types candidates. Between 8 and 26 percent of the data can be classified as potentially/putative a new subsystem. Here samples can be chosen to be investigated further (see supplementary Figure S2).

Conclusion

We developed a method for automatically annotating prokaryote immune systems using Deep Learning. Here, we had to achieve the following two goals for accurately detecting cassettes of immune systems in prokaryotes: 1) to create models that can classify already known types of immune systems and 2) to create a mechanism that allows for the rejection of unrelated proteins. We achieved the first goal using a BOHB-based optimization process over some baseline deep neural architectures, creating an optimized model that produces good classification accuracies in this complex domain. The second goal is achieved in two ways. First, we create a model rejecting proteins dissimilar to proteins known to belong to the immune system. Second, we calibrate our model for classifying immune system proteins to get a reliable confidence measurement for our predictions, which allows us to reject unrelated proteins. For this purpose, we first create a test bench that compares the effect of combining different methods on the mean distance between related and unrelated proteins. This distance is maximized using a combination of Deep Open Classification and temperature scaling. Using the created calibrated model, we can also determine potential candidates for new immune system subtypes using a second cutoff. A clustering mechanism then matches the spatially close immune systems to a cassette. This allows us to scan the genome of archaea, bacteria, and plasmids for immune systems. Given this set of potential new candidate proteins, the obvious next step is to investigate these candidates that potentially belong to a new subtype of the immune system experimentally.

Materials and Methods

Benchmarking uncertainty - calibration

In the past, neural networks were well-calibrated to indicate useful certainties. Despite achieving much better accuracy today, this is only sometimes the case for modern neural networks [33]. [33] also observe that architecture decisions that increase accuracy can decrease calibration. Particular models with a high capacity using batch normalization, little weight decay, and cross-entropy loss tend to be miss-calibrated.

Following the notation from [34], we denote with X an instance in the features space $\mathcal{X}$. A supervised multi-class classification problem for k classes can be stated as finding a probabilistic classifier $\hat{\mathbf{p}} : \mathcal{X} \rightarrow \Delta_k$, yielding a probability vector $\hat{\mathbf{p}}(X) = (\hat{p}_1(X), ..., \hat{p}_k(X))^\top$ for the input $X \in \mathcal{X}$, where $\Delta_k = \{(q_1...q_k)^\top \in [0,1]^k | \sum_{i=1}^{k} q_i = 1\}$. In many other publications, the ith component $\hat{p}_i(X)$ of the probabilistic classifier is denoted as the conditional probability $p_\theta(Y = i \mid X)$, where θ are parameter of the model.

Now in deep neuronal networks, it is common to define the probabilistic classifier via a softmax over the network's *logits* $\mathbf{z}(X) = (z_1(X), ..., z_k(X))^\top$ for a specific instance X. In this case, the pre-

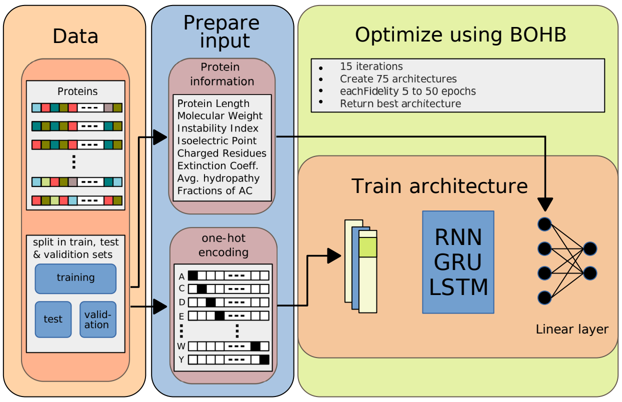

Figure 3. Overview of the training procedure. We separate the data in training, testing, and validation, using 10% of the data for testing and 10% for validation to utilize an early stopping procedure. The proteins are encoded using a one-hot encoding. Additional information can be fed into the linear layers of the used architecture. We optimize this process using BOHB by creating 75 unique architectures in 15 runs for a smaller fidelity. We then tune the resulting best architecture manually on full fidelity. This results in an architecture using a 1d-convolution, which feeds an encoding into a two-layered bidirectional GRU unit. The output of this unit is then concatenated to prepossessed additional information before the final output is created using a linear classifier. To keep the illustration simple we only show three architectures.

dicted probability $\hat{p}_i(X)$ with $i \in \{1, ..., k\}$ is defined as:

$$\hat{p}_i(X) = \sigma_{SM}^{\mathbf{z}(X)}(z_i(X)) =: \frac{\exp(z_i(X))}{\sum_{j=1}^{k} \exp(z_j(X))} \quad (1)$$

In the following, we will write σ_{SM} as short for $\sigma_{SM}^{\mathbf{z}(X)}$ when $\mathbf{z}$ is clear from the context. Furthermore, we will overload the symbol σ_{SM} to also apply it to vectors. Using this, Eq. 1 can simply be written as $\hat{\mathbf{p}}(X) = \sigma_{SM}(\mathbf{z}(X))$. Now given any prediction vector $\mathbf{q} = (q_1...q_k)^\top \in \Delta_k$, the perfect multiclass-calibration is then defined for an probabilistic classifier $\hat{\mathbf{p}}(X)$ as follows [34]:

$$fori = 1, ..., k : \mathbb{P}(Y = i | \hat{\mathbf{p}}(X) = \mathbf{q}) = q_i \quad (2)$$

It simply states that for all instances X with the predicted class probabilities $\mathbf{q}$ (i.e., in the event $\hat{\mathbf{p}}(X) = \mathbf{q}$), the proportion of classes over these instances equals $\mathbf{q}$. Methods to improve the calibration were chosen to be ubiquitously usable and not rely on data augmentation. We decide not to use data augmentation techniques since they implicitly rely on the assumption that the input class is invariant to minor changes. We do not know whether this holds for our dataset. Hence we test the methods temperature, matrix, and vector described in [33] as well as Deep Open Classification (DOC) from [31], penalizing confidence [35] and label smoothing [36, 37] in both single and ensemble use, as shortly described in the following subsections.

Cutoff

The most simplistic idea to identify uncertain class prediction is to create a cutoff for every class we predict.

Penalizing confidence

Now given a deep neuronal network where the probabilistic classifier $\hat{\mathbf{p}}(X)$ is defined using the softmax of the logits (Eq. 1), one problem that leads to miss-calibration are over-confident predictions, which correspond to outputs favoring a particular class for each input X. This corresponds to probability distributions with low entropy. Thus, we can penalize confidence directly using the entropy [35]. The penalty is now activated by adding the entropy,

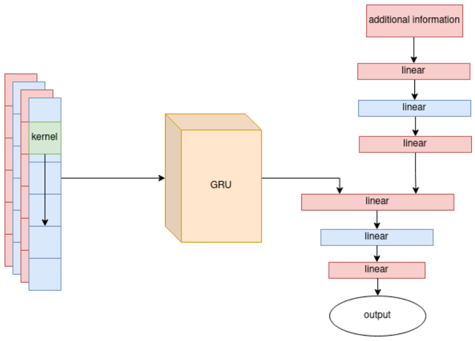

Figure 4. After optimization, the final architecture (with additional information) consists of one convolution layer, two bidirectional GRU layers, and three linear output layers. A second head processes additional information provided by the sequence (see Fig.5). The outcome of this preprocessing is concatenated with the output of the GRU module.

given as

$$H(\hat{\mathbf{p}}(X)) = -\sum_{i=1}^{k} \hat{p}_i(X) \log(\hat{p}_i(X))$$

to the log-likelihood function during training,

$$L = -\sum_{X \in X} \sum_{i=1}^{k} (p_i \log(\hat{p}_i(X))) - \beta H(\hat{\mathbf{p}}(X)),$$

where p_i is the empirical distribution of the ith label in the training set, and β regulates the extent of punishment [35].
To avoid overfitting towards the end of the training process and still enable fast convergence, we can use a hinge loss that penalizes the confidence only when a certain entropy threshold is exceeded [35].

$$L = -\sum_{X \in X} \sum_{i=1}^{k} (p_i \log(\hat{p}_i(X))) - \beta \max(0, \Omega - H(\hat{\mathbf{p}}(X)))$$

Ω stands here for the entropy threshold that the output must exceed.

Deep Open Classification

The Deep Open Classification (DOC) method describes the construction of a multiclass classifier with a 1-vs residual final layer of sigmoids. It uses Gaussian fitting to reduce the free space risk further. For the i-th sigmoid function with class i, the positive examples are all data points with $y = i$, and the negative examples are all data points with $y \neq i$. The output of the ith sigmoid on the input X is then used as predicted probabilities[1]

$$\hat{p}_i(X) = Sigmoid(z_i(X)) \quad (3)$$

The loss of one sample X is then the sum of all log-loss functions of all sigmoids [31]:

$$L = \sum_{i=1}^{k} -I(Y = i) \log(\hat{p}_i(X))$$

1 Please note that the *predicted probabilities* in this case do not form a probability distribution as the probabilities do not sum up to one; we are, however, following here the standard terms in the field as used in e.g. [31]

$$-I(y \neq i)\log(1-\hat{p}_i(X))$$

where I is the indicator function. Note that the probabilistic classifier $\hat{p}_i(X)$ is here defined using the sigmoid function as in Eq. 3, instead of the softmax function. From this follows the classification and rejection procedure:

$$y = \begin{cases} \text{reject} & \text{if } \hat{p}_i(X) < t_i, \forall i \in \{1,...,k\} \\ \underset{i\in\{1,..,k\}}{\text{argmax}}\ \hat{p}_i(X) & \text{otherwise} \end{cases}$$

For each class $i \in \{1,...,k\}$ the limit t_i can be calculated as a Gaussian by using the predicted probabilities of the correctly classified points with the mean μ_i as one half of a Gaussian distribution and constructing the other half by mirroring the points. Using the standard deviation σ of this distribution, [31] then obtain the limit:

$$t_i = max(0.5, 1-\alpha * \sigma)$$

A common choice for α is 3 [31].

Label smoothing

For every example, the model calculates the probability using a softmax function. The loss is then calculated according to [36, 37] as a cross-entropy loss:

$$L = -\sum_{i=1}^{k} \log \hat{p}_i(X) q(Y=i)$$

Here $q(Y)$ is the ground truth distribution. In training, the log-likelihood is maximized for the Dirac delta $q(Y) = \delta_{Y,i}$, which is either 0 when $y \neq i$ or 1. Since fitting to the ground truth can lead to over likelihood, a simple technique for smoothing the labels is proposed. Using a distribution over labels $\mu(Y)$ and a smoothing parameter ϵ:

$$q'(Y=i) = (1-\epsilon)\delta_{y,i} + \epsilon\mu(Y=i)$$

to replace the Dirac delta $q(Y) = \delta_{Y,i}$. The distribution $\mu(Y)$ is created by using the uniform distribution $\mu(Y) \sim 1/k$ with k labels [36, 37].

Matrix and vector scaling - Platt scaling

Matrix and vector scaling are multi-class extensions of Platt scaling. With $\mathbf{z}(X)$ being the logits vector before applying the softmax layer, matrix scaling now applies a linear transformation [33]:

$$\begin{aligned} \mathbf{z}^{scale}(X) &= \mathbf{W}\mathbf{z}(X) + \mathbf{b} \\ \hat{\mathbf{p}}(X) &= \sigma_{SM}(\mathbf{z}^{scale}(X)) \end{aligned}$$

The parameters $\mathbf{W}$ and $\mathbf{b}$ are optimized for the cross-entropy loss on the validation set. Vector scaling uses a vector instead of a matrix [33].

Temperature scaling

Temperature scaling is a version of Platt Logistic Scaling [38] that uses only one parameter. It simply uses a parameter T to rescale logit scores $\mathbf{z}(X)$ before applying the softmax function.

$$\hat{\mathbf{p}}(X) = \sigma_{SM}(\mathbf{z}(X)/T)$$

An optimal T can be received by minimizing the negative log-likelihood on a validation set [38, 33].

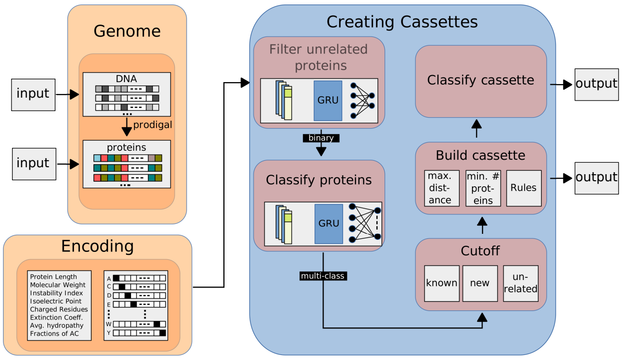

Figure 5. Overview of the classification workflow. The genome is read as nucleotides and translated into proteins using prodigal. Alternatively, proteins can be used as an input. The proteins are encoded in a one-hot fashion, and additional information about the proteins is created. In the first step, these proteins can be filtered using a binary classification model trained on immune system and unrelated proteins. We use a cutoff that favors the prediction of immune system proteins since using cassettes for classification naturally drives down the false-positive rate. The second classification model now classifies the immune systems and allows us to detect new immune types or reject unrelated proteins. Based on the classification, we build possible cassettes and classify the cassette type by allowing users to choose the maximum distance between proteins and the minimum number of proteins belonging to a rule.

Ensembles

In the context of neural networks, ensembles can be constructed to improve prediction quality or uncertainty estimation. Empirically, using ensembles for uncertainty prediction has already shown promising results, possibly because, unlike stochastic models, they can capture different modes of the underlying solution space [39]. [33] observed that the model capacity negatively influences calibration, which also motivates the choice of ensembles since it allows us to use smaller models and still achieve good accuracy. We build our ensembles by randomly initiating each network's parameters and predicting the class with the highest confidence in the averaged output of all ensemble members. We choose the lowest confidence that agrees with the determined class assignment as class certainty to get reliable uncertainty estimations.

General Optimization Strategy

We use BOHB (Bayesian Optimization with Hyperband, see [40]) to optimize eight baseline architectures on a smaller fidelity. This optimization process affects the kernel sizes, stride, number of channels, dropout, and output dimension of the recursive unit. When using an architecture with a second head, we also optimize the number of nodes used to process the data. We additionally optimize a transformer-based architecture for classification, using positional and linear encoding, and optimize the number of heads for the attention layer, dropout, the number of linear layers, and the embedding dimension. The baseline architectures are designed to be relatively shallow to avoid problems with overconfidence in classification [33]. We run 15 iterations for each baseline architecture to create 75 unique, optimized hyperparameter configurations and execute 100 runs. The results for the optimization can be seen in Fig. 1. Following the optimization process, we manually improve the best architecture identified by BOHB on the complete fidelity.

Deepdefense architecture and training

The final architecture uses one convolutional layer with a kernel size of 7 and a stride of 5, followed by two bidirectional GRU layers and three linear layers to produce the models' output. The architecture can be seen in Fig. 3. This structure has also previously

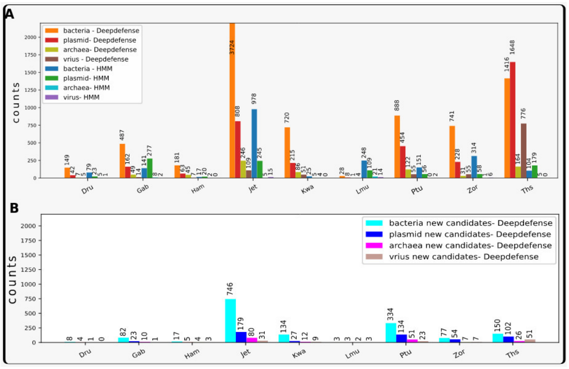

Figure 6. Distribution of immune systems detected using our method and HMM homolog search. Despite the smaller dataset, more immune system candidates were found in bacteria genomes than in plasmids. The lower number of systems belonging to archaea is partially due to the much smaller dataset. Additionally, we did find some cassettes in a bigger phage dataset. The x-axis shows the immune system type, while the y-axis shows the counts of the found immune system cassettes. The barplot (B) shows the number of candidates discovered for further investigation by Deepdefense.

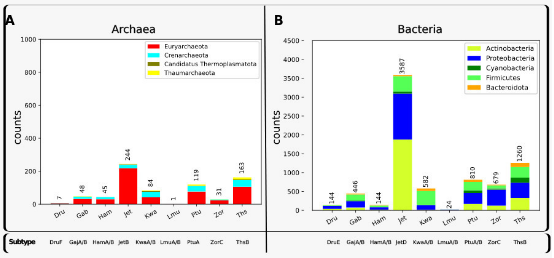

Figure 7. Immune system class distribution for archaea (A) and bacteria (B), color-coded for available phyla. The y-axis shows the counts of the immune cassettes, the x-axis shows the immune system types. In bacteria, we find a majority of detected cassettes belonging to Wadjet, with two phyla particularly prominent. Within archaea, we mostly find cassettes for one phylum.

been shown to create promising results in different classification tasks [41, 30]. The proteins are encoded in a one-hot vector. We use Adam optimizer and a learning rate of 0.001 and train for 150 epochs. After a third of all epochs have passed, we employ early stopping if the validation loss does not improve for 20 epochs to prevent overfitting. We use a MultiStepLearningRateScheduler to adapt our learning rate by a factor of 0.9 after 3, 12, and then every other ten epochs up to 100. In this way, we create three models we use in an ensemble during testing by predicting the class with the highest average confidence and the probability as the lowest model confidence that agrees with this prediction.

Cassette pipeline

The pipeline for the classification of cassettes consists of two distinct modules. The first module classifies proteins as related to the immune system or unrelated. Proteins found to be related can then be passed on to the second module of the pipeline, which performs a multi-class prediction to annotate the specific immune system.

The results of the multi-class prediction can be grouped into different classes depending on two different cutoffs. One cutoff rejects proteins that are considered unrelated to the immune system. The second cutoff determines candidates for a possible later investigation into new immune system types. The resulting classifications are then used to form immune system cassettes according to user specifications.

Availability of Source Code and Requirements

- Project name: Deepdefense
- project home page: https://github.com/SvenHauns/Deepdefense
- Operating system:Platform independent
- Programming language: python
- Other requirements: see public environment file
- publically available under MIT License

Data availability

The data used for training is publically available as part of the publication [26]. The used HMM models are part of the publication [27]. Additionally data was taken from the PADLOC website [32]. The trained model, as well as the additionally created training data, can be found in our GitHub repository.

Additional Files

Supplementary Table S1 The table shows the effect of the chosen combination of methods on the accuracy of the model. In our test bench we find high accuracies for unscaled and temperature scaled methods over all possible combinations and lower accuracies for matrix and vector scaling. This can be explained due to the outcome invariant scaling that temperature scaling use.
Supplementary Table S2 The table shows the class accuracies using the multi-class prediction model based on GRU using additional information executed on 10-fold CV.
Supplementary Figure S1 Distribution of immune systems in archaea and bacteria and plasmids found using our Deepdefense method (blue) vs HMM based homology search (orange) for bacteria (top), archaea (second), plasmids (third), and phage (bottom)
Supplementary Figure S2 Distribution of optimization results using BOHB without additional information. We optimize over the kernel sizes, stride, number of channels, dropout, and output dimension of the recursive unit and over the number of heads for the attention layer, dropout, the number of linear layers, and embedding dimension when using the transformer-based classification model. We can see a slightly higher mean for the GRU-based models. The best architecture here also belongs to GRU models (see outlier)
Supplementary Figure S3 Known immune system proteins (blue) vs. candidates for further investigation (orange) in bacteria. We set a second cutoff based on DOC with an alpha=4 to find candidates that might constitute unknown subtypes.
Supplementary Figure S4 Additionally to optimization without additional information, we also optimize over the number of nodes used to process the additional data. The validation loss is lower for all baseline architectures, using additional information compared to the version without additional information. Again GRU based models do perform best.
Supplementary Figure S5 Overview over distribution for phyla Archaea Eurayarchaeota (A) and Bacteria Proteobacteria with associated classes. The outer circle also shows the distribution o the cassettes found in each class
Supplementary Figure S6 Overview over distribution for phyla Archaea Eurayarchaeota (A) and Bacteria Achinobacteria with associated classes. The outer circle also shows the distribution o the cassettes found in each class

Declarations

List of abbreviations

BOHB: Bayesian Optimization with Hyperband,Doron systems: systems identified in [26], DOC: Deep Open Classification

Consent for publication

Not applicable

Competing Interests

The authors declare that they have no competing interests.

Funding

This work was supported by German Research Foundation (DFG) [BA 2168/23-1/2]; Much more than Defence: the Multiple Functions and Facets of CRISPR–Cas; Baden-Wuerttemberg Ministry of Science, Research and Art; University of Freiburg and also by German Research Foundation (DFG grant BA 2168/25-1 Einfluss von RNA-bindenden Proteinen und mRNA-Strukturen auf alternative Translation-Regulationsmechanismen im entzündlichen Tumorgeschehen)

Author's Contributions

S.H. developed the software and wrote the initial draft of the manuscript. O.S.A. conceived the study. O.S.A., and R.B. oversaw the project. All authors reviewed, contributed to, and approved the manuscript.

Acknowledgements

The authors acknowledge support by the High Performance and Cloud Computing Group at the Zentrum für Datenverarbeitung of the University of Tübingen, the state of Baden-Württemberg through bwHPC and the German Research Foundation (DFG) through grant no INST 37/935-1 FUGG. The authors thank the anonymous reviewers for their valuable suggestions.

References

1. Rohwer F, Thurber RV. Viruses manipulate the marine environment. Nature 2009 May;459:207–212.
2. Suttle C. Viruses in the sea. Nature 2005 10;437:356–361.
3. Suttle C. Marine viruses—Major players in the global ecosystem. Nature reviews Microbiology 2007 11;5:801–812.
4. Edwards RA, Rohwer F. Viral metagenomics. Nat Rev Microbiol 2005 Jun;3:504–510.
5. Hampton HG, Watson BNJ, Fineran PC. The arms race between bacteria and their phage foes. Nature 2020 January;577(7790):327—336. https://doi.org/10.1038/s41586-019-1894-8.
6. Labrie SJ, Samson JE, Moineau S. Bacteriophage resistance mechanisms. Nat Rev Microbiol 2010;8:317–327.
7. Makarova KS, Anantharaman V, Aravind EVK. Live virus-free or die: coupling of antivirus immunity and programmed suicide or dormancy in prokaryotes. Biol Direct 2012;7:40.
8. Makarova KS, Wolf YI, Koonin EV. Comparative genomics of defense systems in archaea and bacteria. Nucleic Acids Res 2013;41(8):4360–4377.
9. Bernheim A, Sorek R. The pan-immune system of bacteria: antiviral defence as a community resource. Nat Rev Microbiol 2020;18:113–119.
10. Anzalone A, Koblan L, Liu D. Genome editing with CRISPR–Cas nucleases, base editors, transposases and prime editors. Nature Biotechnology 2020 06;38:1–21.
11. Hegge J, Swarts D, Oost J. Prokaryotic Argonaute proteins: novel genome-editing tools? Nature Reviews Microbiology 2017 07;16.
12. Loenen WAM, Dryden DTF, Raleigh EA, Wilson GG, Murray NE. Highlights of the DNA cutters: a short history of the restriction enzymes. Nucleic Acids Research 2013 10;42(1):3–19. https://doi.org/10.1093/nar/gkt990.
13. Abby SS, Néron B, Ménager H, Touchon M, Rocha EPC. MacSyFinder: A Program to Mine Genomes for Molecular Systems with an Application to CRISPR-Cas Systems. PLOS ONE 2014 10;9(10):1–9. https://doi.org/10.1371/journal.pone.0110726.
14. Biswas A, Staals RHJ, Morales SE, Fineran PC, Brown CM. CRISPRDetect: A flexible algorithm to define CRISPR arrays. BMC Genomics 2016;17:356. https://doi.org/10.1186/s12864-016-2627-0.
15. Couvin D, Bernheim A, Toffano-Nioche C, Touchon M, Michalik J, Néron B, et al. CRISPRCasFinder, an update of CRISRFinder, includes a portable version, enhanced performance and integrates search for Cas proteins. Nucleic Acids Research 2018 05;46(W1):246–251. https://doi.org/10.1093/nar/gky425.
16. Crawley AB, Henriksen JR, Barrangou R. CRISPRdisco: An Automated Pipeline for the Discovery and Analysis of CRISPR-Cas Systems. The CRISPR Journal 2018;1(2):171–181. https://doi.org/10.1089/crispr.2017.0022, pMID: 31021201.
17. Grissa I, Vergnaud G, Pourcel C. CRISPRFinder: a web tool to identify clustered regularly interspaced short palindromic repeats. Nucleic Acids Res 2007 07;35.
18. Padilha VA, Alkhnbashi OS, Shah SA, de Carvalho ACPLF, Backofen R. CRISPRcasIdentifier: Machine learning for accurate identification and classification of CRISPR-Cas systems. GigaScience 2020 06;9(6). https://doi.org/10.1093/gigascience/giaa062, giaa062.
19. Russel J, Pinilla-Redondo R, Mayo-Muñoz D, Shah SA, Sørensen SJ. CRISPRCasTyper: Automated Identification, Annotation, and Classification of CRISPR-Cas Loci. CRISPR J 2020 Dec;3(6):462–469.
20. Padilha VA, Alkhnbashi OS, Tran VD, Shah SA, Carvalho ACPLF, Backofen R. Casboundary: automated definition of integral Cas cassettes. Bioinformatics 2020 11;https://doi.org/10.1093/bioinformatics/btaa984, btaa984.
21. Mitrofanov A, Alkhnbashi OS, Shmakov SA, Makarova KS, Koonin EV, Backofen R. CRISPRidentify: identification of CRISPR arrays using machine learning approach. Nucleic Acids Res 2021 Feb;49(4):e20.
22. Alkhnbashi OS, Mitrofanov A, Bonidia R, Raden M, Tran VD, Eggenhofer F, et al. CRISPRloci: comprehensive and accurate annotation of CRISPR-Cas system. Nucleic Acids Res 2021;.
23. Mitrofanov A, Ziemann M, Alkhnbashi OS, Hess WR, Backofen R. CRISPRtracrRNA: robust approach for CRISPR tracrRNA detection. Bioinformatics 2022 09;38(Supplement 2):42–48. https://doi.org/10.1093/bioinformatics/btac466.
24. Koonin EV, S MK, I WY. Evolutionary genomics of defense systems in archaea and bacteria. Annu Rev Microbiol 2017;71:233–261.
25. Makarova KS, Wolf YI, Snir S, Koonin EV. Defense Islands in Bacterial and Archaeal Genomes and Prediction of Novel Defense Systems. Journal of Bacteriology 2011;193(21):6039–6056. https://journals.asm.org/doi/abs/10.1128/JB.05535-11.
26. Doron S, Melamed S, Ofir G, Leavitt A, Lopatina A, Keren M, et al. Systematic discovery of antiphage defense systems in the microbial pangenome. Science 2018;359(6379):eaar4120. https://www.science.org/doi/abs/10.1126/science.aar4120.
27. Payne L, Todeschini T, Wu Y, Perry B, Ronson C, Fineran P, et al. Identification and classification of antiviral defence systems in bacteria and archaea with PADLOC reveals new system types. Nucleic acids research 2021 10;49.
28. Fei G, Liu B. Breaking the Closed World Assumption in Text

Classification. In: Proceedings of the 2016 Conference of the North American Chapter of the Association for Computational Linguistics: Human Language Technologies San Diego, California: Association for Computational Linguistics; 2016. p. 506–514. https://aclanthology.org/N16-1061.
29. Byrd J, Lipton ZC. What is the Effect of Importance Weighting in Deep Learning? In: International Conference on Machine Learning; 2018. .
30. Wandera KG, Alkhnbashi OS, v I Bassett H, Mitrofanov A, Hauns S, Migur A, et al. Anti-CRISPR prediction using deep learning reveals an inhibitor of Cas13b nucleases. Molecular Cell 2022;82(14):2714–2726.e4. https://www.sciencedirect.com/science/article/pii/S1097276522004373.
31. Shu L, Xu H, Liu B. DOC: Deep Open Classification of Text Documents. In: Proceedings of the 2017 Conference on Empirical Methods in Natural Language Processing Copenhagen, Denmark: Association for Computational Linguistics; 2017. p. 2911–2916. https://www.aclweb.org/anthology/D17-1314.
32. Leighton PJ, Meaden S, Mestre MR, Palmer C, Toro N, Fineran PC, et al. PADLOC: a web server for the identification of antiviral defence systems in microbial genomes. Nucleic Acids Research 2022;50:W541–W550.
33. Guo C, Pleiss G, Sun Y, Weinberger KQ. On Calibration of Modern Neural Networks. CoRR 2017;abs/1706.04599. http://arxiv.org/abs/1706.04599.
34. Kull M, Perello-Nieto M, Kängsepp M, de Menezes e Silva Filho T, Song H, Flach PA. Beyond temperature scaling: Obtaining well-calibrated multiclass probabilities with Dirichlet calibration. In: Neural Information Processing Systems; 2019. .
35. Pereyra G, Tucker G, Chorowski J, Kaiser L, Hinton GE. Regularizing Neural Networks by Penalizing Confident Output Distributions. CoRR 2017;abs/1701.06548. http://arxiv.org/abs/1701.06548.
36. Szegedy C, Vanhoucke V, Ioffe S, Shlens J, Wojna Z. Rethinking the Inception Architecture for Computer Vision. CoRR 2015;abs/1512.00567. http://arxiv.org/abs/1512.00567.
37. Müller R, Kornblith S, Hinton GE. When Does Label Smoothing Help? CoRR 2019;abs/1906.02629. http://arxiv.org/abs/1906.02629.
38. Platt J. Probabilistic Outputs for Support Vector Machines and Comparisons to Regularized Likelihood Methods. Adv Large Margin Classif 2000 06;10.
39. Lakshminarayanan B, Pritzel A, Blundell C. Simple and Scalable Predictive Uncertainty Estimation using Deep Ensembles. Information Processing Systems 2017;p. 6402–6413.
40. Falkner S, Klein A, Hutter F. BOHB: Robust and Efficient Hyperparameter Optimization at Scale. CoRR 2018;abs/1807.01774. http://arxiv.org/abs/1807.01774.
41. Zhang JX, Yordanov B, Gaunt A, Wang WX, Dai P, Chen YJ, et al. A deep learning model for predicting next-generation sequencing depth from DNA sequence. Nature Communications 2021;12:4387.

supplementary

Click here to access/download
Supplementary Material
supplementary.pdf
