# Supplementary material for: Deepdefense: annotation of immune systems in prokaryotes using deep learning
Source: Gigascience. 2024 Oct 10;13:giae062. doi: 10.1093/gigascience/giae062 (PMC11959188; doi:10.1093/gigascience/giae062)
Supplement: giae062_GIGA-D-23-00300_Revision_2 [file giae062_giga-d-23-00300_revision_2.pdf]

GigaScience

Deepdefense: Annotation of immune systems in prokaryotes using DeepLearning

--Manuscript Draft--

**Manuscript Number:**	GIGA-D-23-00300R2
**Full Title:**	Deepdefense: Annotation of immune systems in prokaryotes using DeepLearning
**Article Type:**	Technical Note
**Funding Information:**	Deutsche Forschungsgemeinschaft (BA 2168/23-1/) — Professor Rolf Backofen<br>Deutsche Forschungsgemeinschaft (BA 2168/25-1) — Professor Rolf Backofen
**Abstract:**	Background Due to a constant evolutionary arms race, archaea and bacteria have evolved an abundance and diversity of immune responses to protect themselves against phages. Since the discovery and application of CRISPR-Cas adaptive immune systems, numerous novel candidates for immune systems have been identified. Previous approaches to identifying these new immune systems rely on HMM-based homolog searches or use labor-intensive and costly wet-lab experiments. To aid in finding and classifying immune systems, we use machine learning to classify already known immune system proteins and discover potential candidates in the genome. Neural networks have shown promising results in classifying and predicting protein functionality in recent years. However, these approaches frequently rely on the assumption of a closed world, which is often different in practice.<br>Results In this work, we explore neural networks for immune protein classification and deal with different methods for rejecting unrelated proteins in a genome-wide search, and establish a benchmark. Then, we optimize our approach for accuracy. Based on this, we develop an algorithm called Deepdefense to predict immune cassette classes based on a genome. Finally, we test our approach for detecting immune systems in the genome against an HMM-based method.<br>Conclusions Deepdefense can automatically detect genes and define cassette annotations and classifications using two model classifications. This is achieved by creating an optimized deep learning model to annotate immune systems, in combination with calibration methods, and a second model to enable the scanning of an entire genome.
**Corresponding Author:**	Sven Hauns<br>Albert-Ludwigs-Universität Freiburg: Albert-Ludwigs-Universitat Freiburg<br>Freiburg, GERMANY
**Corresponding Author Secondary Information:**	
**Corresponding Author's Institution:**	Albert-Ludwigs-Universität Freiburg: Albert-Ludwigs-Universitat Freiburg
**Corresponding Author's Secondary Institution:**	
**First Author:**	Sven Hauns
**First Author Secondary Information:**	
**Order of Authors:**	Sven Hauns<br>Omer Alkhnbashi<br>Rolf Backofen
**Order of Authors Secondary Information:**	
**Response to Reviewers:**	responses to the revision is included in the main manuscript file.

Powered by Editorial Manager® and ProduXion Manager® from Aries Systems Corporation

Additional Information:	
**Question**	**Response**
Are you submitting this manuscript to a special series or article collection?	No
**Experimental design and statistics**<br><br>Full details of the experimental design and statistical methods used should be given in the Methods section, as detailed in our Minimum Standards Reporting Checklist. Information essential to interpreting the data presented should be made available in the figure legends.<br><br>Have you included all the information requested in your manuscript?	Yes
**Resources**<br><br>A description of all resources used, including antibodies, cell lines, animals and software tools, with enough information to allow them to be uniquely identified, should be included in the Methods section. Authors are strongly encouraged to cite Research Resource Identifiers (RRIDs) for antibodies, model organisms and tools, where possible.<br><br>Have you included the information requested as detailed in our Minimum Standards Reporting Checklist?	Yes
**Availability of data and materials**<br><br>All datasets and code on which the conclusions of the paper rely must be either included in your submission or deposited in publicly available repositories (where available and ethically appropriate), referencing such data using a unique identifier in the references and in the "Availability of Data and Materials" section of your manuscript.	Yes

Powered by Editorial Manager® and ProduXion Manager® from Aries Systems Corporation

Have you have met the above requirement as detailed in our Minimum Standards Reporting Checklist?	

Manuscript

Click here to access/download;Manuscript;response_to_reviewers_Deepdef

Dear Dr. Nogoy
Editor-in-Chief of GiGascience

Herewith we submit the revised version of our manuscript “Deepdefense: Annotation of immune systems in prokaryotes using Deep Learning”.

We have answered each point and have modified our submission accordingly. We have also carefully considered the reviewer’s comments and integrated them into a thoroughly revised version of the manuscript.

We thank the reviewers for their supportive comments and constructive feedback and the editor for the opportunity to submit a revised manuscript. Our response to each comment below is in blue, while the corresponding changes to the main text are in green.

Additionally we added the bio.tools and SciCrunch.org identifiers to the manuscript.

I am looking forward to hearing from you.

Sincerely yours,
Prof. Rolf Backofen

Point-to-point response to reviewer comments:

1. Please make the following statement in the Abstract section clearer:
However, these approaches frequently rely on the assumption of a closed world, which is often different in practice.

I saw there was a reference for this statement in the Introduction section, but please make it more readable here to readers.

Author's response
We thank the reviewer for his comment.We augmented the abstract to clarify the statement.

2. Please double check the following statement in the Introduction section:
As the own DNA is methylated, this protects any unmethylated foreign DNA.

Author's response
Thank you for the comment. We corrected the error in the statement.

3. The following two sentences in the Introduction section are repetitive - please consider re-organising them:

We choose to employ deep learning with the expectation that the automatic generation of intricate features will enable us to identify proteins even with lower structural similarity to already known samples. We opt for deep learning, anticipating that its capacity for automatically generating intricate features will enable the identification of proteins, even those with lower structural similarity to known samples.

Author's response
Thank you very much. We removed the redundancy in the text.

4. In the Introduction section, it says:
We create twelve different features characterizing the used proteins (e.g. protein length, instability index) as in [30]. The full list can be found in the supplementary Table S1.

But Table S1 only shows 8 protein properties. Please clarify this.

Author’s response
We appreciate the reviewer's point. The original table summarized some of the 12 properties into one bullet point. We improved the table now to explicitly mention all 12 protein properties.

5. The title of Figure S3 writes "Additionally to optimization without additional information". "Without" or "With"? Please double check.

Author’s response
Thank you. We changed the statement to clarify its meaning.

6. Please consider updating Figures 4, 6 & 7 in high-quality - current versions are hard to read.

Author’s response
We thank the reviewer for his comment. We improved the figures by providing greater legend and label sizes and replaced them with higher quality versions.

GigaScience, 2023, 1–10

doi: xx.xxxx/xxxx
Manuscript in Preparation
Paper

PAPER

Deepdefense: Annotation of immune systems in prokaryotes using Deep Learning

Sven Hauns[1], Omer S. Alkhnbashi[2,3,*] and Rolf Backofen[1,4,*]

[1]Bioinformatics group,Department of Computer Science, University of Freiburg,Georges-Köhler-Allee 101,79110,Germany and [2]Center for Applied and Translational Genomics (CATG), Mohammed Bin Rashid University of Medicine and Health Sciences, Dubai Healthcare City, Dubai, United Arab Emirates and [3]College of Medicine, Mohammed Bin Rashid University of Medicine and Health Sciences, Dubai Healthcare City, Dubai, United Arab Emirates and [4]Signalling Research Centres BIOSS and CIBSS, University of Freiburg, Schänzlestr. 18, 79104 Freiburg, Germany

[*]Corresponding authors: omer.Alkhnbashi@mbru.ac.ae, backofen@informatik.uni-freiburg.de

Abstract

Background Due to a constant evolutionary arms race, archaea and bacteria have evolved an abundance and diversity of immune responses to protect themselves against phages. Since the discovery and application of CRISPR-Cas adaptive immune systems, numerous novel candidates for immune systems have been identified. Previous approaches to identifying these new immune systems rely on HMM-based homolog searches or use labor-intensive and costly wet-lab experiments. To aid in finding and classifying immune systems genomes, we use machine learning to classify already known immune system proteins and discover potential candidates in the genome. Neural networks have shown promising results in classifying and predicting protein functionality in recent years. However, these methods often operate under the closed world assumption, where it is presumed that all potential outcomes or classes are already known and included in the training dataset. This assumption doesn't always hold true in real-world scenarios, such as in genomics, where new samples can emerge that were not previously accounted for in the training phase.
Results In this work, we explore neural networks for immune protein classification and deal with different methods for rejecting unrelated proteins in a genome-wide search, and establish a benchmark. Then, we optimize our approach for accuracy. Based on this, we develop an algorithm called Deepdefense to predict immune cassette classes based on a genome. This design facilitates the differentiation between immune system-related and unrelated proteins by analyzing variations in model-predicted confidence values, aiding in the identification of both known and potentially novel immune system proteins. Finally, we test our approach for detecting immune systems in the genome against an HMM-based method.
Conclusions Deepdefense can automatically detect genes and define cassette annotations and classifications using two model classifications. This is achieved by creating an optimized deep learning model to annotate immune systems, in combination with calibration methods, and a second model to enable the scanning of an entire genome.

Key words:
immune systems; deep learning; classification; genome

Introduction

Viruses are the most abundant biological entities in biospheres such as soil and sea, vastly outnumbering prokaryotes [1, 2, 3, 4]. Given such an abundance of numbers, phages, and plasmids frequently attack bacteria and archaea. Because of such attacks and the resulting arms race, they have evolved various techniques to defend themselves [5, 6].

Compiled on: May 20, 2024.
Draft manuscript prepared by the author.

Key Points

- We develop a deep learning based approach for the classification of Doron immune systems in whole genomes based on two classifiers.
- The first classifier filters proteins most likely not belonging to the immune system, while the second classifier classifies immune system sub-classes or rejects unrelated data.
- The resulting output classification is then used to build specific cassettes for each immune system type.
- We improve the model calibration using different methods to reject unrelated data reliably.
- Using the calibrated model, we also suggest candidates which may constitute a new immune system.

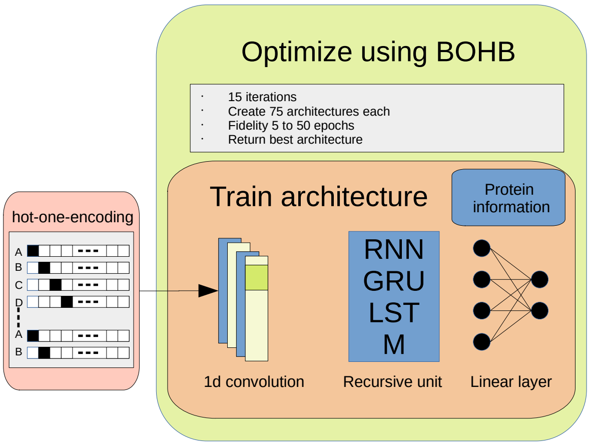

Figure 1. Overview of the training procedure. We separate the data in training, testing, and validation, using 10% of the data for testing and 10% for validation to utilize an early stopping procedure. The proteins are encoded using a one-hot encoding. Additional information can be fed into the linear layers of the used architecture. We optimize this process using BOHB by creating 75 unique architectures in 15 runs for a smaller fidelity. We then tune the resulting best architecture manually on full fidelity. This results in an architecture using a 1d-convolution, which feeds an encoding into a two-layered bidirectional GRU unit. The output of this unit is then concatenated to prepossessed additional information before the final output is created using a linear classifier. To keep the illustration simple we only show three architectures.

These techniques can be grouped into two general defense mechanisms based on the principle of their action: 1) innate or adaptive immunity, and 2) programmed cell death and dormancy [7, 8]. Innate immunity is often based on restriction-modification (R-M) systems, where a restriction enzyme cuts unmethylated DNA.

As the own DNA is methylated, this protects against any unmethylated foreign DNA [6].

In contrast, the CRISPR-Cas systems provide adaptive immunity, which recognizes and degrades specific viral nucleic acids [9]. The ability of the CRISPR systems to precisely cut and integrate viral nucleic acids into the bacteria genome has led to the creation of important new genetic tools, such as Cas9 for gene editing [10, 11, 12]).

The success of CRISPR/Cas9 has fuelled the search for new members of the CRISPR-Cas systems and led to many different computational approaches, both scoring or machine learning (ML) based, to identify new members of the CRISPR-Cas system [13, 14, 15, 16, 17, 18, 19, 20, 21, 22, 23]. However, no ML approach exists to date to identify immune systems different from CRISPR, and to suggest candidates for further exploration.

We opt for deep learning, anticipating that its capacity for automatically generating intricate features will enable the identification of proteins, even those with lower structural similarity to known samples.

The ability of deep learning models to recognize and leverage subtle patterns for classification should empower it to capture and comprehend complex relationships, thereby enhancing their effectiveness in annotation.

To set up such an ML approach for non-CRISPR immune systems, we have to ask ourselves whether there is some chance to detect an immune system in some prokaryotic genome. The estimated proportion of genes coding for immune systems varies from about 0.0001% to 10% [24]. The given distributions for each defense system show the lower limit because many more variants need to be identified due to the rapid mutation of defense genes. The number of defense genes in a genome shows a linear scaling with the size of the genome [8]. The genomes responsible for immune response are often close together and form so-called defense islands [25]. In the following, we would like to exploit this property to predict immune cassettes based on gene predictions ultimately. Our approach focuses on identifying immune systems identified in [26], named Druantia, Gabija, Hachiman, Kiwa, Lamassu, Septu, Shedu, Thoeris, Wadjet, and Zorya. Following [27], these systems are called the 'Doron systems.'

In this work, we present a deep learning approach called Deep-Defense, designed to capture much of the relevant information used in manual annotation. It is purely based on features for different protein sequences related to immune systems. The proposed approach addresses the problem of annotating and classifying Doron defense gene systems into types and subtypes by using evidence from a series of consecutive genes that form part of a cassette to identify the various proteins involved. This approach enables us to represent the genomics of anti-viral systems as cassettes of adjacent proteins encoded in the genome. As our features for the deep learning approach correspond to evidence for immune-related protein sequences, we can determine proteins whose evidence is critical for predicting types and subtypes. Since we want to use our tool to scan the entire genome, we need to be able to reject unrelated proteins reliably. We try to improve the problems caused by this closed-world assumption [28] by using techniques designed to improve model calibration. Furthermore, we compare this approach with the recently published approach based on the HMM (hidden markov model) homology search [27] and demonstrate that Deepdefense can identify more immune systems in prokaryotic genomes. Additionally, the tool identifies potential new immune systems related to the Doron systems.

Data Description

Our dataset comprises 21,196 unique validated samples [26]. To ensure ample training data for subclasses with limited samples, we employ a stratified 10-fold cross-validation split, allocating 10% of the dataset for testing in each cross-validation step, and another

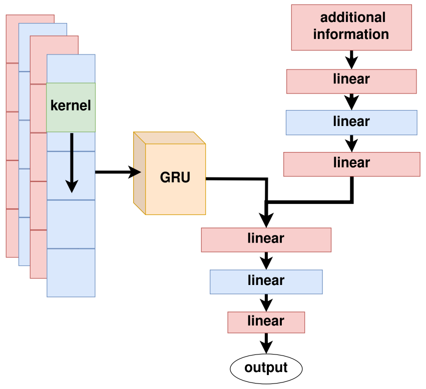

Figure 2. After optimization, the final architecture (with additional information) consists of one convolution layer, two bidirectional GRU layers, and three linear output layers. A second head processes additional information provided by the sequence (see supplementary Table S1). The outcome of this preprocessing is concatenated with the output of the GRU module.

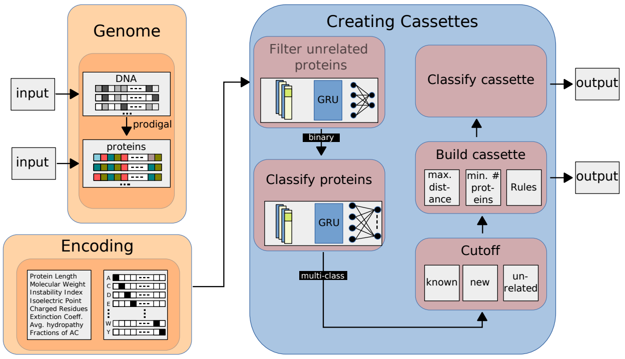

Figure 3. Overview of the classification workflow. The genome is read as nucleotides and translated into proteins using prodigal. Alternatively, proteins can be used as an input. The proteins are encoded in a one-hot fashion, and additional information about the proteins is created. In the first step, these proteins can be filtered using a binary classification model trained on immune system and unrelated proteins. We use a cutoff that favors the prediction of immune system proteins since using cassettes for classification naturally drives down the false-positive rate. The second classification model now classifies the immune systems and allows us to detect new immune types or reject unrelated proteins. Based on the classification, we build possible cassettes and classify the cassette type by allowing users to choose the maximum distance between proteins and the minimum number of proteins belonging to a rule.

10% of the remaining dataset as a validation set for early stopping. Due to sampling in a stratified fashion a representative number of samples for each class is contained in each test set. This results in 17,169 samples for training, 1,908 for validation, and 2,119 for testing in each cross-validation split. Additionally, we use BLAST to create datasets with varying degrees of sequence homology (95%, 90%, 80%, 70%, 60%, 50%, 40% and 30%), and we also report our results on these sets.

The dataset we used was imbalanced for types, consisting of 1263 Durantia samples, 3723 Gajiba samples,1529 Hachiman samples, 6882 Wadjet samples, 637 Lamassu samples, 2807 Septu samples, 647 Shedu samples, 1097 Thoeris samples, 745 Kiwa samples, 1866 Zorya samples. Hence there were only roughly one-tenth of the samples of Lamassu than for Wadjet. We weight the samples during training according to their distribution in the dataset to prevent overfitting to majority classes [29], by dividing the total number of samples by the number of samples in a class multiplied by the number of classes. Additionally, we use a dataset with 21196 samples from bacteria and archaea that have an unrelated function to the immune system, also ensuring a low similarity to known defense system by sequence homology and HMM based search. In addition to cross-validation, we also use 10 % of the data for an independent test dataset with a maximal sequence homoglogy of 95. We create twelve different features characterizing the used proteins (e.g. protein length, instability index) as in [30]. The full list can be found in the supplementary Table S1.

Materials and Methods

Benchmarking uncertainty - calibration

In the past, neural networks were well-calibrated to indicate useful certainties. Despite achieving much better accuracy today, this is only sometimes the case for modern neural networks [31]. [31] also observe that architecture decisions that increase accuracy can decrease calibration. Particular models with a high capacity using batch normalization, little weight decay, and cross-entropy loss tend to be miss-calibrated.

Following the notation from [32], we denote with X an instance in the features space $\mathcal{X}$. A supervised multi-class classification problem for k classes can be stated as finding a probabilistic classifier $\hat{\mathbf{p}}$: $\mathcal{X} \rightarrow \Delta_k$, yielding a probability vector $\hat{\mathbf{p}}(X) = (\hat{p}_1(X), ..., \hat{p}_k(X))^\top$ for the input $X \in \mathcal{X}$, where $\Delta_k = \{(q_1...q_k)^\top \in [0,1]^k | \sum_{i=1}^{k} q_i = 1\}$. In many other publications, the ith component $\hat{p}_i(X)$ of the probabilistic classifier is denoted as the conditional probability $p_\theta(Y = i \mid X)$, where θ are parameter of the model.

Now in deep neuronal networks, it is common to define the probabilistic classifier via a softmax over the network's *logits* $\mathbf{z}(X) = (z_1(X), ..., z_k(X))^\top$ for a specific instance X. In this case, the predicted probability $\hat{p}_i(X)$ with $i \in \{1, ..., k\}$ is defined as:

$$\hat{p}_i(X) = \sigma_{SM}^{\mathbf{z}(X)}(z_i(X)) =: \frac{\exp(z_i(X))}{\sum_{j=1}^{k} \exp(z_j(X))} \tag{1}$$

In the following, we will write σ_{SM} as short for $\sigma_{SM}^{\mathbf{z}(X)}$ when $\mathbf{z}$ is clear from the context. Furthermore, we will overload the symbol σ_{SM} to also apply it to vectors. Using this, Eq. 1 can simply be written as $\hat{\mathbf{p}}(X) = \sigma_{SM}(\mathbf{z}(X))$. Now given any prediction vector $\mathbf{q} = (q_1...q_k)^\top \in \Delta_k$, the perfect multiclass-calibration is then defined for an probabilistic classifier $\hat{\mathbf{p}}(X)$ as follows [32]:

$$for\ i = 1, ..., k : \mathbb{P}(Y = i | \hat{\mathbf{p}}(X) = \mathbf{q}) = q_i \tag{2}$$

It simply states that for all instances X with the predicted class probabilities $\mathbf{q}$ (i.e., in the event $\hat{\mathbf{p}}(X) = \mathbf{q}$), the proportion of classes over these instances equals $\mathbf{q}$. Methods to improve the calibration were chosen to be ubiquitously usable and not rely on data augmentation. We decide not to use data augmentation techniques since they implicitly rely on the assumption that the input class is invariant to minor changes. We do not know whether this holds for our dataset. Hence we test the methods *temperature*, *matrix*, and *vector* described in [31] as well as *Deep Open Classification (DOC)* from [33], *label smoothing* [34, 35] and *penalizing confidence* [36] in both single and ensemble use, as shortly described in the following subsections.

Cutoff

The most simplistic idea to identify uncertain class prediction is to create a cutoff for every class we predict.

Penalizing confidence

Now given a deep neuronal network where the probabilistic classifier $\hat{\mathbf{p}}(X)$ is defined using the softmax of the logits (Eq. 1), one problem that leads to miss-calibration are over-confident predictions, which correspond to outputs favoring a particular class for each input X. This corresponds to probability distributions with low entropy. Thus, we can penalize confidence directly using the entropy [36]. The penalty is now activated by adding the entropy,

Table 1. Benchmark for neural networks in single use

method	softmax		DOC[1]		smoothing		penalize		**method dist.**		**method acc.**	
scaling	dist.	acc.	dist.	acc.	dist.	acc.	dist.	acc.	mean	sd	mean	sd
unscaled	0.10	0.99	0.18	0.99	0.11	0.97	0.12	0.99	0.13	0.0329	0.99	0.0079
temp scaling	0.12	0.99	0.20	0.99	0.14	0.97	0.14	0.99	0.15	0.0306	0.99	0.0079
vector scaling	0.15	0.80	-0.14	0.55	0.21	0.96	0.10	0.75	0.08	0.1321	0.77	0.1461
matrix scaling	0.15	0.55	0.02	0.63	0.17	0.39	0.14	0.64	0.12	0.0562	0.55	0.0984
**scaling mean**	0.13	0.83	0.07	0.79	0.16	0.83	0.12	0.84				
**scaling sd**	0.022	0.179	0.138	0.202	0.036	0.251	0.015	0.155				

Table 2. Benchmark for neural networks in ensemble use

method	softmax		DOC[1]		smoothing		penalize		**method dist.**		**method acc.**	
scaling	dist.	acc.	dist.	acc.	dist.	acc.	dist.	acc.	mean	sd	mean	sd
unscaled	0.10	0.99	0.29	1.0	0.11	0.99	0.11	1.0	0.15	0.0777	1.0	0.0045
temp scaling	0.13	0.99	**0.30**	1.0	0.14	0.99	0.14	1.0	0.18	0.0691	1.0	0.0045
vector scaling	0.10	0.97	0.15	0.64	0.14	0.76	0.12	0.78	0.13	0.0184	0.79	0.1202
matrix scaling	0.13	0.58	**0.30**	0.74	0.11	0.78	0.18	0.87	0.18	0.0757	0.74	0.1053
**scaling mean**	0.12	0.88	0.26	0.84	0.12	0.88	0.14	0.91				
**scaling sd**	0.014	0.175	0.064	0.161	0.014	0.109	0.026	0.092				

The tables show the average distance between related and unrelated proteins for a combination of methods relying on scaling or modifying the training process. Results are given for both single (using only one neural network, table 1) and ensemble (using the output of three neural networks, table 2) use. We see the best results for the DOC method in ensemble use in combination with temperature and matrix scaling. Here, we observe an average difference of 30 % in classification probabilities between proteins unrelated to the immune system and those related to the immune system, which allows us to clearly distinguish the two different classes and will be used in the subsequent analysis. We furthermore noted that the use of an ensemble outperforms the single use in most cases when measuring the distance and, in all cases, when determining the accuracy. We additionally assess the overall effectiveness of the scaling approach and calibration method employed. Our findings reveal that temperature scaling exerts the biggest influence, without adversely affecting the overall accuracy. For ensemble use DOC showed the best over all performance, displaying moderate variation over different scaling methods, as indicated by the corresponding standard deviation.
[1]DOC: Deep Open Classification.

given as

$$H(\hat{\mathbf{p}}(X)) = -\sum_{i=1}^{k} \hat{p}_i(X)\log(\hat{p}_i(X))$$

to the log-likelihood function during training,

$$L = -\sum_{X\in X}\sum_{i=1}^{k}(p_i \log(\hat{p}_i(X))) - \beta H(\hat{\mathbf{p}}(X)),$$

where p_i is the empirical distribution of the ith label in the training set, and β regulates the extent of punishment [36].
To avoid overfitting towards the end of the training process and still enable fast convergence, we can use a hinge loss that penalizes the confidence only when a certain entropy threshold is exceeded [36].

$$L = -\sum_{X\in X}\sum_{i=1}^{k}(p_i \log(\hat{p}_i(X))) - \beta \max(0, \Omega - H(\hat{\mathbf{p}}(X)))$$

Ω stands here for the entropy threshold that the output must exceed.

Deep Open Classification

The Deep Open Classification (DOC) method describes the construction of a multiclass classifier with a 1-vs residual final layer of sigmoids. It uses Gaussian fitting to reduce the free space risk further. For the i-th sigmoid function with class i, the positive examples are all data points with $y = i$, and the negative examples are all data points with $y \neq i$. The output of the ith sigmoid on the input X is then used as predicted probabilities[1]

$$\hat{p}_i(X) = Sigmoid(z_i(X)) \tag{3}$$

The loss of one sample X is then the sum of all log-loss functions of all sigmoids [33]:

$$L = \sum_{i=1}^{k} -I(Y = i)\log(\hat{p}_i(X))$$

$$-I(y \neq i)\log(1 - \hat{p}_i(X))$$

where I is the indicator function. Note that the probabilistic classifier $\hat{p}_i(X)$ is here defined using the sigmoid function as in Eq. 3, instead of the softmax function. From this follows the classification and rejection procedure:

$$y = \begin{cases} \text{reject} & \text{if } \hat{p}_i(X) < t_i, \forall i \in \{1, ..., k\} \\ \underset{i\in\{1,..,k\}}{\text{argmax}}\ \hat{p}_i(X) & \text{otherwise} \end{cases}$$

For each class $i \in \{1, ..., k\}$ the limit t_i can be calculated as a Gaussian by using the predicted probabilities of the correctly classified points with the mean μ_i as one half of a Gaussian distribution and constructing the other half by mirroring the points. Using the standard deviation σ of this distribution, [33] then obtain the limit:

$$t_i = max(0.5, 1 - \alpha * \sigma)$$

1 Please note that the *predicted probabilities* in this case do not form a probability distribution as the probabilities do not sum up to one; we are, however, following here the standard terms in the field as used in e.g. [33]

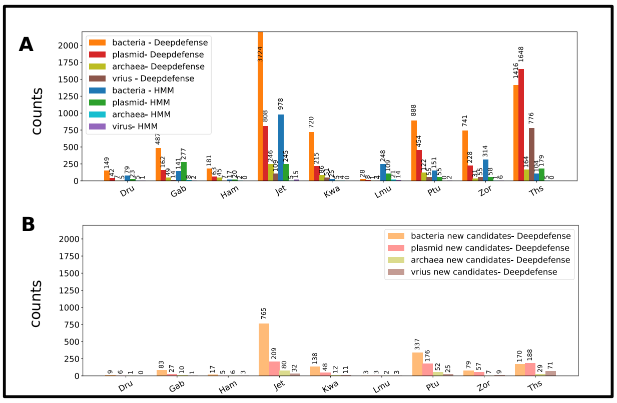

Figure 4. Distribution of immune systems detected using our method and HMM homolog search. Despite the smaller dataset, more immune system candidates were found in bacteria genomes than in plasmids. The lower number of systems belonging to archaea is partially due to the much smaller dataset. Additionally, we did find some cassettes in a bigger phage dataset. The x-axis shows the immune system type, while the y-axis shows the counts of the found immune system cassettes. The barplot (B) shows the number of candidates discovered for further investigation by Deepdefense.

We adhere to the methodology of the original publication by selecting α as 3. According to [33], this choice functions as an outlier detection measure, as it represents the number of standard deviations a point must be from the mean.

Label smoothing

For every example, the model calculates the probability using a softmax function. The loss is then calculated according to [34, 35] as a cross-entropy loss:

$$L = -\sum_{i=1}^{k} \log \hat{p}_i(X) q(Y = i)$$

Here $q(Y)$ is the ground truth distribution. In training, the log-likelihood is maximized for the Dirac delta $q(Y) = \delta_{Y,i}$, which is either 0 when $y \neq i$ or 1. Since fitting to the ground truth can lead to over likelihood, a simple technique for smoothing the labels is proposed. Using a distribution over labels $\mu(Y)$ and a smoothing parameter ϵ:

$$q'(Y = i) = (1 - \epsilon)\delta_{y,i} + \epsilon\mu(Y = i)$$

to replace the Dirac delta $q(Y) = \delta_{Y,i}$. The distribution $\mu(Y)$ is created by using the uniform distribution $\mu(Y) \sim 1/k$ with k labels [34, 35].

Matrix and vector scaling - Platt scaling

Deepdefense architecture and training

Matrix and vector scaling are multi-class extensions of Platt scaling. With $\mathbf{z}(X)$ being the logits vector before applying the softmax layer, matrix scaling now applies a linear transformation [31]:

$$\begin{aligned} \mathbf{z}^{scale}(X) &= \mathbf{W}\mathbf{z}(X) + \mathbf{b} \\ \hat{\mathbf{p}}(X) &= \sigma_{SM}(\mathbf{z}^{scale}(X)) \end{aligned}$$

The parameters $\mathbf{W}$ and $\mathbf{b}$ are optimized for the cross-entropy loss on the validation set. Vector scaling uses a vector instead of a matrix [31].

Temperature scaling

Temperature scaling is a version of Platt Logistic Scaling [37] that uses only one parameter. It simply uses a parameter T to rescale logit scores $\mathbf{z}(X)$ before applying the softmax function.

$$\hat{\mathbf{p}}(X) = \sigma_{SM}(\mathbf{z}(X)/T)$$

An optimal T can be received by minimizing the negative log-likelihood on a validation set [37, 31].

Ensembles

In the context of neural networks, ensembles can be constructed to improve prediction quality or uncertainty estimation. Empirically, using ensembles for uncertainty prediction has already shown promising results, possibly because, unlike stochastic models, they can capture different modes of the underlying solution space [38]. [31] observed that the model capacity negatively influences calibration, which also motivates the choice of ensembles since it allows us to use smaller models and still achieve good accuracy. We build our ensembles by randomly initiating each network's parameters and predicting the class with the highest confidence in the averaged output of all ensemble members. We choose the lowest confidence that agrees with the determined class assignment as class certainty to get reliable uncertainty estimations.

General Optimization Strategy

We use BOHB (Bayesian Optimization with Hyperband, see [39]) to optimize eight baseline architectures on a smaller fidelity. This optimization process affects the kernel sizes, stride, number of channels, dropout, and output dimension of the recursive unit. When using an architecture with a second head, we also optimize the number of nodes used to process the data. The basic structure of this process can be seen in Fig. 1. We additionally optimize a transformer-based architecture for classification, using positional and linear encoding, and optimize the number of heads for the attention layer, dropout, the number of linear layers, and the embedding dimension. The baseline architectures are designed to be relatively shallow to avoid problems with overconfidence in classification [31]. We run 15 iterations for each baseline architecture to create 75 unique, optimized hyperparameter configurations and execute 100 runs. Following the optimization process, we manually improve the best architecture identified by BOHB on the complete fidelity.

The final architecture uses one convolutional layer with a kernel size of 7 and a stride of 5, followed by two bidirectional GRU layers and three linear layers to produce the models' output. The architecture can be seen in Fig. 2. This structure has also previously been shown to create promising results in different classification tasks [40, 30]. The proteins are encoded in a one-hot vector. We use Adam optimizer and a learning rate of 0.001 and train for 150 epochs. After a third of all epochs have passed, we employ early stopping if the validation loss does not improve for 20 epochs to prevent overfitting. We use a MultiStepLearningRateScheduler to adapt our learning rate by a factor of 0.9 after 3, 12, and then every other ten epochs up to 100. In this way, we create three models we use in an ensemble during testing by predicting the class with the highest average confidence and the probability as the lowest model confidence that agrees with this prediction.

Cassette pipeline

The pipeline for the classification of cassettes consists of two distinct modules. The first module classifies proteins as related to the immune system or unrelated. Proteins found to be related can then

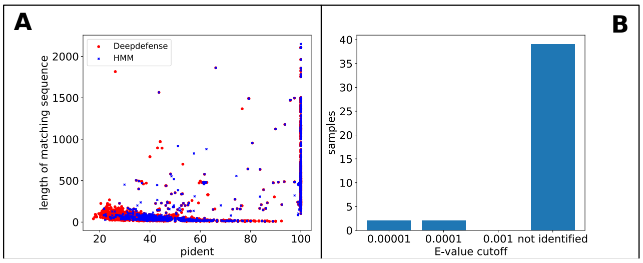

Figure 5. Presented is a scatterplot (A) illustrating the relationship between pident (percentage of identical positions) and the length of matching sequences for all proteins within a cassette identified by both Deepdefense (10335 samples) and Hidden Markov Model (2348 samples) in bacterial genomes. It is noteworthy that there is significant overlap between the two methods; however, Deepdefense excels in detecting proteins with lower sequence similarity. The values for pident and the length of matching sequences were generated using BLAST, which mapped annotated proteins against the original dataset used for training purposes. The bar plot on the right (B) depicts the distribution of E-values for sequences with moderate pident (>40, <50) and matching sequence length (>30), where we also identify neighboring proteins associated with immune system cassettes. Despite significant sequence homology and appropriate neighborhood alignment, a notable number of proteins remain unidentified by the existing HMM-based approach.

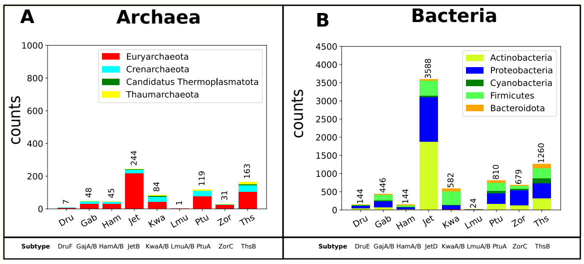

Figure 6. Immune system class distribution for archaea (A) and bacteria (B), color-coded for available phyla. The y-axis shows the counts of the immune cassettes, the x-axis shows the immune system types. In bacteria, we find a majority of detected cassettes belonging to Wadjet, with two phyla particularly prominent. Within archaea, we mostly find cassettes for one phylum.

be passed on to the second module of the pipeline, which performs a multi-class prediction to annotate the specific immune system.

The results of the multi-class prediction can be grouped into different classes depending on two different cutoffs. One cutoff rejects proteins that are considered unrelated to the immune system. The second cutoff determines candidates for a possible later investigation into new immune system types. The resulting classifications are then used to form immune system cassettes according to user specifications.

This design enables us to discern disparities between proteins associated and unassociated with the immune system by evaluating variations in the confidence values predicted by the model. Furthermore, this approach can serve as a valuable tool in distinguishing between proteins already identified as part of the immune system and those that may potentially represent novel additions to our understanding of immune system proteins.

Prediction Quality after Optimization

To create a model for our classification scheme, we search over 75 optimized architectures for eight basic architectures. Fig. 1 also shows the optimization process. This search shows a similar distribution for all eight models (see supplementary Figure S2/S3). The best result overall models were found with the GRU-based model. The validation loss for models with additional information is considerably better than those without additional information. Using this model as a baseline, we optimize it on the whole fidelity. The resulting architecture can be seen in Fig. 2. The transformer-based model probably underperforms here due to some classes having a low number of samples. Training and testing this architecture against immune-system-related and immune-system-unrelated proteins in a 5-fold CV, we achieve an accuracy of 0.96 and an excellent ROC-AUC of 0.99. Using a maximal sequence homology of 95 we achieve an accuracy of 0.91 and ROC-AUC of 0.96. To test the quality of our architecture, we employ a 10-fold CV and achieve an average accuracy of 0.96 with an average ROC-AUC of 0.995 and and weighted AUPRC of 0.979 (results for all classes can be seen in the supplementary Table S2).

Our model undergoes testing across diverse sequence homology levels. At maximal homology (95) between proteins we find a high accuracy (0.95), ROC-AUC (0.992), and weighted AUPRC (0.973). Maintaining consistently high performance, at 90 homology, accuracy remains at 0.95 with ROC-AUC (0.994) and weighted AUPRC (0.969). For a homology of 80 we still get a high accuracy (0.93) and good ROC-AUC (0.980) and AUPRC (0.943) values. However, at 70 homology, accuracy slightly decreases to 0.90 with ROC-AUC (0.978) and AUPRC (0.91). Homology of 60 maintains accuracy at 0.90, ROC-AUC (0.982), and AUPRC (0.90). At 50 homology, accuracy is 0.83, AUPRC (0.839), and ROC-AUC (0.973). Opting for homology of 60 for DruA and DruB due to sample limitations. Homology of 40, with only 19 classes having sufficient samples, yields an average accuracy of 0.77, ROC-AUC (0.918), and AUPRC (0.755), while the remaining classes are created at higher homology. In the last run at 30 homology, only JetA and JetC have enough samples, resulting in an average accuracy of JetA and JetC of 0.5, expected

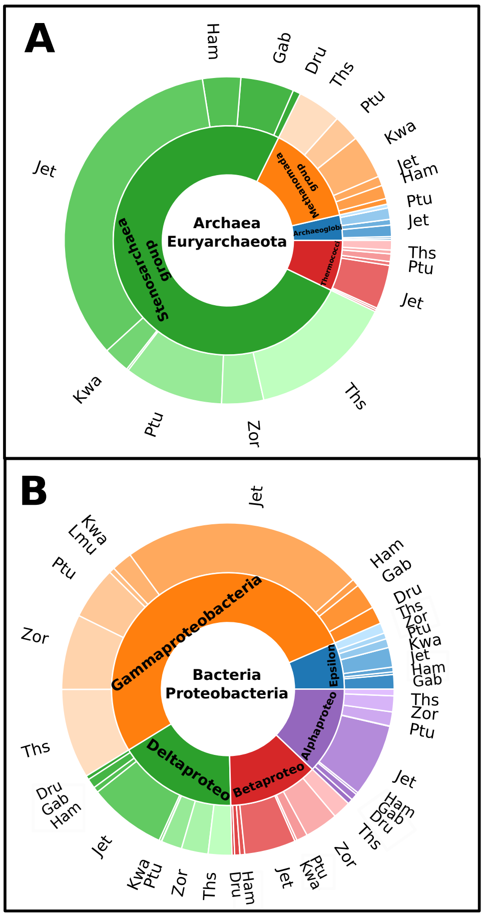

Figure 7. The distribution of detected cassette classes in 3 phyla for multiple classes. Phyla Euryarchaeota (A) with classes Stenosarcaea group, Archeaoglobi, Thermococci, and Methanomado group, and Proteobacteria (B) with classes Gammaproteobacteria, Deltaproteobacteria, Betaproteobacteria, Alphaproteobacteria, Epsilonproteobacteria. The outer circle then shows the distribution of the immune system cassettes.

due to the very low number of training samples. All other classes at this cutoff were created at a higher homology.

Since our ultimate goal is to predict immune system cassettes, which consist of closely clustered and ordered immune system genes, we get a reduced false-positive rate compared to single-gene classification. Therefore we can set the cutoff used for the first module at 0.3, which maximizes the accuracy of correctly identified proteins belonging to the immune system. Next, we use our calibrated network and set the first cutoff of the second module as defined in [33] to $\alpha = 3$, and our second cutoff to $\alpha = 4$. The resulting pipeline can also be seen in Fig. 3.

Results

Benchmark

Since our method should be able to scan the entire genome and reject unrelated proteins, we explore methods to improve the uncertainty estimations. Details on these methods used for the following benchmark are given in the method section. To create a benchmark we first train models on the Zorya class for all calibration methods that do not rely on scaling. Then we determine the mean distance of the prediction between the test set and a set of unrelated proteins using 5-fold CV. In the next step, this unrelated class is used to scale the model logits using temperature, matrix, or vector scaling. The result of this benchmark can be seen in table 1) and 2). This allows us to test the method's usefulness directly on the main task to be improved: the rejection of unrelated proteins in a genome-wide search. The highest mean distances were achieved using a combination of scaling (temperature or matrix) with DOC (Deep Open Classification). We did not find a systematical advantage of label smoothing over using softmax in our test bench. For all techniques, we found an improvement in accuracy in using ensembles compared to single networks. Since temperature scaling multiplies the output with a single parameter, it does not change the order between the values for the class prediction, which is not the case for matrix and vector scaling. Here accuracies may drop (see supplementary Table S3). Due to stable accuracies and an otherwise high mean distance (of 0.30, which is on par with the results achieved by matrix scaling), we choose temperature scaling combined with DOC to scale our methods.

Detecting cassettes in bacteria, archea and plasmids and phage

Forming cassettes of related proteins in the genome exploits the property of immune system proteins to cluster close to each other in so-called defense islands. We choose a maximal distance of two genes between classes belonging to a cassette rule to detect possible cassettes. Since the immune system Shedu is not known to form a cassette, we exclude it from the search. This distance considers the differences in the location of genes belonging to a system. It allows for some variability while using the cluster forming of immune systems. We set the minimum number of genes belonging to a system for identification to two since many cassettes only have up to two belonging candidates. These parameters can be set customarily. We run our algorithm against a database of 18441 plasmids, 799 archaea, 7308 bacteria, and a phage dataset of 2689 samples. The search yielded 3765 candidates in plasmids, with 562 candidates belonging to a potentially new type. For bacteria, we find 8751 candidates and 1608 potentially new immune system cassettes, with a ratio of 0.18. We find a higher ratio of 0.25 in archaea, with 771 known and 195 potentially new types. Within the phage dataset, we find 1078 known types, which mostly belonged to the Thoeris type. The resulting distribution for all discovered cassette types for our Deepdefense approach and the results for an HMM-based search can be seen in Fig.˜4a. Both distributions show a spike for Wadjet-based and Thoeris-based cassettes, while fewer immune system cassettes were found in plasmids, despite the larger dataset (probably due to the smaller genome size). The HMM and our Deepdefense method discover some cassettes in Virus genomes. This might give a hint to the potential origin of the immune system. We also discover potential candidates for currently unknown immune system proteins using our second cutoff. Fig.˜4b shows the relatively low number of potential candidates discovered by Deepdefense. We find a small spike for the Wadjet immune system again and, surprisingly, for Septu.

To conduct a comparison between proteins identified by DeepDefense and those identified through an HMM-based approach, we align the predicted proteins with the training dataset and analyze the percentage identity (pident) and the length of homologous sub-sequences, as depicted in 5. The resulting scatter plot reveals a substantial and anticipated overlap among highly homologous sequences. Notably, DeepDefense predicts a larger set of sequences related to the immune system across a spectrum of homology levels, including both highly homologous and less homologous sequences. This is particularly noticeable when discerning sequences with lower homology levels. Furthermore, we employ sequences featuring neighboring proteins identified as affiliated with immune system cassettes and examine the E-value distribution for sequences exhibiting reasonably high homology and matching sequence length in 5. Despite substantial sequence homology and suitable neighborhood alignment, a considerable number of proteins remain unidentified by a HMM based approach.

Additionally, we conducted a comparative analysis of the outputs generated by the HMM-based approach PADLOC [27, 41] and Deepdefense, focusing on 76 genomes sourced from the PADLOC website. Deepdefense identified a total of 885 proteins associated with defense systems, whereas PADLOC's prediction encompassed 199 proteins affiliated with the Doron systems. Notably, an intersection of 55 proteins was observed, underscoring the distinct cassette analysis approaches employed by both methods. Among the pool of 144 prospective candidates for novel immune systems, PADLOC classified a mere five as systems unrelated to the Doron framework.

Distribution of detected immune systems in three phyla

Using the distribution of phyla for all immune systems (as seen in Fig. 6), we choose the highest expressed phyla and plot the distribution in Fig.˜7. A major phylum of Archaea was Euryarchaeota, with the majority classes Stenosarcaea, Archeaoglobi, Thermococci, and Methanomado and a total number of 535 elements. Three hundred ninety-nine immune cassettes were identified in Stenosarchaea, 19 in Archaeoglobi, 38 in Thermococci, and 75 in Methanomado. For bacteria, we identify two major phyla, Actinobacteria (with a total number of 2642 cassettes, see supplementary Figure S5) and Proteobacteria (with a total number of 2785 cassettes). The former consists mostly of the classes Bifidobacteriales (265 cassettes), Micrococcales (214 cassettes), Streptomycetales(791 cassettes), and Corynebacteriales (585 cassettes), the latter of the classes Gammaproteobacteria (1449 cassettes), Deltaproteobacteria (335 cassettes), Betaproteobacteria (461 cassettes), Alphaproteobacteria (347 cassettes),
Epsilonproteobacteria (180 cassettes).

Conclusion

We developed a method for automatically annotating prokaryote immune systems using Deep Learning. Here, we had to achieve the following two goals for accurately detecting cassettes of immune systems in prokaryotes: 1) to create models that can classify already known types of immune systems and 2) to create a mechanism that

allows for the rejection of unrelated proteins. We achieved the first goal using a BOHB-based optimization process over some baseline deep neural architectures, creating an optimized model that produces good classification accuracies in this complex domain. The second goal is achieved in two ways. First, we create a model rejecting proteins dissimilar to proteins known to belong to the immune system. Second, we calibrate our model for classifying immune system proteins to get a reliable confidence measurement for our predictions, which allows us to reject unrelated proteins. For this purpose, we first create a test bench that compares the effect of combining different methods on the mean distance between related and unrelated proteins. This distance is maximized using a combination of Deep Open Classification and temperature scaling. Using the created calibrated model, we can also determine potential candidates for new immune system subtypes using a second cutoff. A clustering mechanism then matches the spatially close immune systems to a cassette. This allows us to scan the genome of archaea, bacteria, and plasmids for immune systems. Given this set of potential new candidate proteins, the obvious next step is to investigate these candidates that potentially belong to a new subtype of the immune system experimentally.

Availability of Source Code and Requirements

- Project name: Deepdefense
- project home page: https://github.com/SvenHauns/Deepdefense
- Operating system:Platform independent
- Programming language: python
- Other requirements: see public environment file
- publically available under MIT License

Data availability

The data used for training is publically available as part of the publication [26]. The used HMM models are part of the publication [27]. Additionally data was taken from the PADLOC website [41]. The trained model, as well as the additionally created training data, can be found in our GitHub repository.

The tool is registered under the ID SCR_025346 on SciCrunch.org and as deepdefense in the bio.tools database.

Additional Files

Supplementary Table S1 The table shows the additional protein information computed for the second model input.
Supplementary Table S2 The table shows the class accuracies using the multi-class prediction model based on GRU using additional information executed on 10-fold CV.
Supplementary Table S3 The table shows the effect of the chosen combination of methods on the accuracy of the model. In our test bench we find high accuracies for unscaled and temperature scaled methods over all possible combinations and lower accuracies for matrix and vector scaling. This can be explained due to the outcome invariant scaling that temperature scaling use.
Supplementary Figure S1 A more detailed description of the final architecture used for classification.
Supplementary Figure S2 Distribution of optimization results using BOHB without additional information. We optimize over the kernel sizes, stride, number of channels, dropout, and output dimension of the recursive unit and over the number of heads for the attention layer, dropout, the number of linear layers, and embedding dimension when using the transformer-based classification model. We can see a slightly higher mean for the GRU-based models. The best architecture here also belongs to GRU models (see outlier)
Supplementary Figure S3 Additionally to optimization without additional information, we also optimize over the number of nodes used to process the additional data. The validation loss is lower for all baseline architectures, using additional information compared to the version without additional information. Again GRU based models do perform best.
Supplementary Figure S4 Overview over distribution for phyla Archaea Eurayarchaeota (A) and Bacteria Proteobacteria with associated classes. The outer circle also shows the distribution o the cassettes found in each class
Supplementary Figure S5 Overview over distribution for phyla Archaea Eurayarchaeota (A) and Bacteria Achinobacteria with associated classes. The outer circle also shows the distribution o the cassettes found in each class

Declarations

List of abbreviations

BOHB: Bayesian Optimization with Hyperband,Doron systems: systems identified in [26], DOC: Deep Open Classification

Consent for publication

Not applicable

Competing Interests

The authors declare that they have no competing interests.

Funding

Deutsche Forschungsgemeinschaft Grant, BA 2168/23, Much more than Defence: the Multiple Functions and Facets of CRISPR–Cas. And Deutsche Forschungsgemeinschaft, Grant, BA 2168/25-1, Einluss von RNA-bindenden Proteinen und mRNA-Strukturen auf alternative Translation-Regulationsmechanismen im entzündlichen Tumorgeschehen. The article processing charge is funded by the Baden-Wuerttemberg Ministry of Science, Research and Art and the University of Freiburg in the funding programme Open Access Publishing

Author's Contributions

S.H. developed the software and wrote the initial draft of the manuscript. O.S.A. conceived the study. O.S.A., and R.B. oversaw the project. All authors reviewed, contributed to, and approved the manuscript.

Acknowledgements

The authors acknowledge support by the High Performance and Cloud Computing Group at the Zentrum für Datenverarbeitung of the University of Tübingen, the state of Baden-Württemberg through bwHPC and the German Research Foundation (DFG) through grant no INST 37/935-1 FUGG. This work was supported by the BMBF-funded de.NBI Cloud within the German Network for Bioinformatics Infrastructure (031A532B, 031A533A, 031A533B, 031A534A, 031A535A, 031A537A, 031A537B, 031A537C, 031A537D, 031A538A). The authors thank the reviewers for their valuable suggestions.

References

1. Rohwer F, Thurber RV. Viruses manipulate the marine environment. Nature 2009 May;459:207–212.
2. Suttle C. Viruses in the sea. Nature 2005 10;437:356–361.
3. Suttle C. Marine viruses—Major players in the global ecosystem. Nature reviews Microbiology 2007 11;5:801–812.
4. Edwards RA, Rohwer F. Viral metagenomics. Nat Rev Microbiol 2005 Jun;3:504–510.
5. Hampton HG, Watson BNJ, Fineran PC. The arms race between bacteria and their phage foes. Nature 2020 January;577(7790):327—336. https://doi.org/10.1038/s41586-019-1894-8.
6. Labrie SJ, Samson JE, Moineau S. Bacteriophage resistance mechanisms. Nat Rev Microbiol 2010;8:317–327.
7. Makarova KS, Anantharaman V, Aravind EVK. Live virus-free or die: coupling of antivirus immunity and programmed suicide or dormancy in prokaryotes. Biol Direct 2012;7:40.
8. Makarova KS, Wolf YI, Koonin EV. Comparative genomics of defense systems in archaea and bacteria. Nucleic Acids Res 2013;41(8):4360–4377.
9. Bernheim A, Sorek R. The pan-immune system of bacteria: antiviral defence as a community resource. Nat Rev Microbiol 2020;18:113–119.
10. Anzalone A, Koblan L, Liu D. Genome editing with CRISPR–Cas nucleases, base editors, transposases and prime editors. Nature Biotechnology 2020 06;38:1–21.
11. Hegge J, Swarts D, Oost J. Prokaryotic Argonaute proteins: novel genome-editing tools? Nature Reviews Microbiology 2017 07;16.
12. Loenen WAM, Dryden DTF, Raleigh EA, Wilson GG, Murray NE. Highlights of the DNA cutters: a short history of the restriction enzymes. Nucleic Acids Research 2013 10;42(1):3–19. https://doi.org/10.1093/nar/gkt990.
13. Abby SS, Néron B, Ménager H, Touchon M, Rocha EPC. MacSyFinder: A Program to Mine Genomes for Molecular Systems with an Application to CRISPR-Cas Systems. PLOS ONE 2014 10;9(10):1–9. https://doi.org/10.1371/journal.pone.0110726.
14. Biswas A, Staals RHJ, Morales SE, Fineran PC, Brown CM. CRISPRDetect: A flexible algorithm to define CRISPR arrays. BMC Genomics 2016;17:356. https://doi.org/10.1186/s12864-016-2627-0.
15. Couvin D, Bernheim A, Toffano-Nioche C, Touchon M, Michalik J, Néron B, et al. CRISPRCasFinder, an update of CRISRFinder, includes a portable version, enhanced performance and integrates search for Cas proteins. Nucleic Acids Research 2018 05;46(W1):246–251. https://doi.org/10.1093/nar/gky425.
16. Crawley AB, Henriksen JR, Barrangou R. CRISPRdisco: An Automated Pipeline for the Discovery and Analysis of CRISPR-Cas Systems. The CRISPR Journal 2018;1(2):171–181. https://doi.org/10.1089/crispr.2017.0022, pMID: 31021201.
17. Grissa I, Vergnaud G, Pourcel C. CRISPRFinder: a web tool to identify clustered regularly interspaced short palindromic repeats. Nucleic Acids Res 2007 07;35.
18. Padilha VA, Alkhnbashi OS, Shah SA, de Carvalho ACPLF, Backofen R. CRISPRcasIdentifier: Machine learning for accurate identification and classification of CRISPR-Cas systems. GigaScience 2020 06;9(6). https://doi.org/10.1093/gigascience/giaa062, giaa062.
19. Russel J, Pinilla-Redondo R, Mayo-Muñoz D, Shah SA, Sørensen SJ. CRISPRCasTyper: Automated Identification, Annotation, and Classification of CRISPR-Cas Loci. CRISPR J 2020 Dec;3(6):462–469.
20. Padilha VA, Alkhnbashi OS, Tran VD, Shah SA, Carvalho ACPLF, Backofen R. Casboundary: automated definition of integral Cas cassettes. Bioinformatics 2020 11;https://doi.org/10.1093/bioinformatics/btaa984, btaa984.
21. Mitrofanov A, Alkhnbashi OS, Shmakov SA, Makarova KS, Koonin EV, Backofen R. CRISPRidentify: identification of CRISPR arrays using machine learning approach. Nucleic Acids Res 2021 Feb;49(4):e20.
22. Alkhnbashi OS, Mitrofanov A, Bonidia R, Raden M, Tran VD, Eggenhofer F, et al. CRISPRloci: comprehensive and accurate annotation of CRISPR-Cas system. Nucleic Acids Res 2021;.
23. Mitrofanov A, Ziemann M, Alkhnbashi OS, Hess WR, Backofen R. CRISPRtracrRNA: robust approach for CRISPR tracrRNA detection. Bioinformatics 2022 09;38(Supplement 2):42–48. https://doi.org/10.1093/bioinformatics/btac466.
24. Koonin EV, S MK, I WY. Evolutionary genomics of defense systems in archaea and bacteria. Annu Rev Microbiol 2017;71:233–261.
25. Makarova KS, Wolf YI, Snir S, Koonin EV. Defense Islands in Bacterial and Archaeal Genomes and Prediction of Novel Defense Systems. Journal of Bacteriology 2011;193(21):6039–6056. https://journals.asm.org/doi/abs/10.1128/JB.05535-11.
26. Doron S, Melamed S, Ofir G, Leavitt A, Lopatina A, Keren M, et al. Systematic discovery of antiphage defense systems in the microbial pangenome. Science 2018;359(6379):eaar4120. https://www.science.org/doi/abs/10.1126/science.aar4120.
27. Payne L, Todeschini T, Wu Y, Perry B, Ronson C, Fineran P, et al. Identification and classification of antiviral defence systems in bacteria and archaea with PADLOC reveals new system types. Nucleic acids research 2021 10;49.
28. Fei G, Liu B. Breaking the Closed World Assumption in Text Classification. In: Proceedings of the 2016 Conference of the North American Chapter of the Association for Computational Linguistics: Human Language Technologies San Diego, California: Association for Computational Linguistics; 2016. p. 506–514. https://aclanthology.org/N16-1061.
29. Byrd J, Lipton ZC. What is the Effect of Importance Weighting in Deep Learning? In: International Conference on Machine Learning; 2018. .
30. Wandera KG, Alkhnbashi OS, v I Bassett H, Mitrofanov A, Hauns S, Migur A, et al. Anti-CRISPR prediction using deep learning reveals an inhibitor of Cas13b nucleases. Molecular Cell 2022;82(14):2714–2726.e4. https://www.sciencedirect.com/science/article/pii/S1097276522004373.
31. Guo C, Pleiss G, Sun Y, Weinberger KQ. On Calibration of Modern Neural Networks. CoRR 2017;abs/1706.04599. http://arxiv.org/abs/1706.04599.
32. Kull M, Perello-Nieto M, Kängsepp M, de Menezes e Silva Filho T, Song H, Flach PA. Beyond temperature scaling: Obtaining well-calibrated multiclass probabilities with Dirichlet calibration. In: Neural Information Processing Systems; 2019. .
33. Shu L, Xu H, Liu B. DOC: Deep Open Classification of Text Documents. In: Proceedings of the 2017 Conference on Empirical Methods in Natural Language Processing Copenhagen, Denmark: Association for Computational Linguistics; 2017. p. 2911–2916. https://www.aclweb.org/anthology/D17-1314.
34. Szegedy C, Vanhoucke V, Ioffe S, Shlens J, Wojna Z. Rethinking the Inception Architecture for Computer Vision. CoRR 2015;abs/1512.00567. http://arxiv.org/abs/1512.00567.
35. Müller R, Kornblith S, Hinton GE. When Does Label Smoothing Help? CoRR 2019;abs/1906.02629. http://arxiv.org/abs/1906.02629.
36. Pereyra G, Tucker G, Chorowski J, Kaiser L, Hinton GE. Regularizing Neural Networks by Penalizing Confident Output Distributions. CoRR 2017;abs/1701.06548. http://arxiv.org/abs/1701.06548.
37. Platt J. Probabilistic Outputs for Support Vector Machines and Comparisons to Regularized Likelihood Methods. Adv Large Margin Classif 2000 06;10.
38. Lakshminarayanan B, Pritzel A, Blundell C. Simple and Scalable Predictive Uncertainty Estimation using Deep Ensembles.

Information Processing Systems 2017;p. 6402–6413.
39. Falkner S, Klein A, Hutter F. BOHB: Robust and Efficient Hyperparameter Optimization at Scale. CoRR 2018;abs/1807.01774. http://arxiv.org/abs/1807.01774.
40. Zhang JX, Yordanov B, Gaunt A, Wang WX, Dai P, Chen YJ, et al. A deep learning model for predicting next-generation sequencing depth from DNA sequence. Nature Communications 2021;12:4387.
41. Leighton PJ, Meaden S, Mestre MR, Palmer C, Toro N, Fineran PC, et al. PADLOC: a web server for the identification of antiviral defence systems in microbial genomes. Nucleic Acids Research 2022;50:W541–W550.

protein information
Protein Length
Molecular Weight
Isoelectric Point
Negative charged residues
positive charged residues
Extinction Coefficient 1
Extinction Coefficient 2
Instability Index
Average of hydropathy
Fractions of AC

Table 1. The table shows the additional protein information computed for the second model input.

	average	JetA	JetB	JetC	JetD	ZorA	ZorB	ZorC	ZorD	ZorE	KwaA
accuracy		0.97	0.99	0.98	0.97	0.98	0.98	0.94.	0.92	0.95	0.99
	KwaB	GajA	GajB	HamA	HamB	LmuA	LmuB	SduA	PtuA	PtuB	ThsA
accuracy	0.94	0.96	0.97	0.96	0.95	0.92	0.96	0.93	0.97	0.99	0.89
	ThsB	DruA	DruB	DruC	DruD	DruE	DruF	DruG	DruH	DruM	
accuracy	0.94	0.93	0.84	0.73	0.92	0.98	0.83	0.90	0.93	0.94	

Table 2. The table shows the class accuracies using the multi-class prediction model based on GRU using additional information executed on 10-fold CV.

	single use				ensemble use			
scaling	softmax	DOC	smoothing	punish	softmax	DOC	smoothing	punish
unscaled	0.99	0.99	0.97	0.99	0.99	1.0	0.99	1.0
temp scaled	0.99	0.99	0.97	0.99	0.99	1.0	0.99	1.0
vector scaling	0.8	0.55	0.96	0.75	0.97	0.64	0.76	0.78
matrix scaling	0.55	0.63	0.39	0.64	0.58	0.74	0.78	0.87

Table 3. The table shows the effect of the chosen combination of methods on the accuracy of the model. In our test bench we find high accuracies for unscaledand temperature scaled methods over all possible combinations and lower accuracies for matrix and vector scaling. This can be explained due to the outcome invariant scaling that temperature scaling uses.

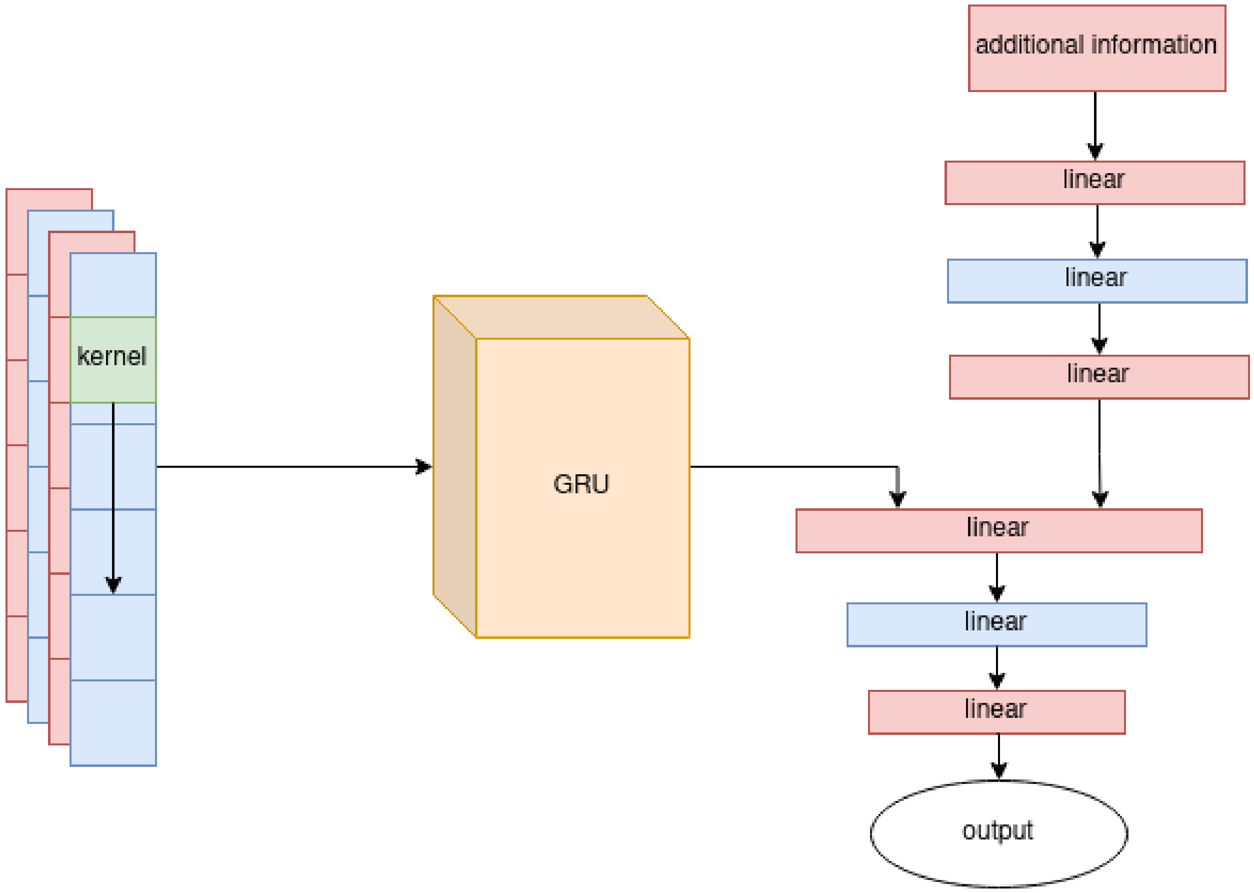

Figure 1. The model processes the input first using a 1D convolution with 45 output channel and a kernel size of 7 and a stride of 5. The following bidirectional GRU unit with 20 output channel, 2 layers and roughly a dropout of 0.23. The output is decreased to the classification layer by three linear layers, first to quarter, then to a eight, then to to size of the output layer. The protein information is preprocessed by three linear layer with a hidden dimension of 71. Between all linear layer we make use of 1d batch normalization.

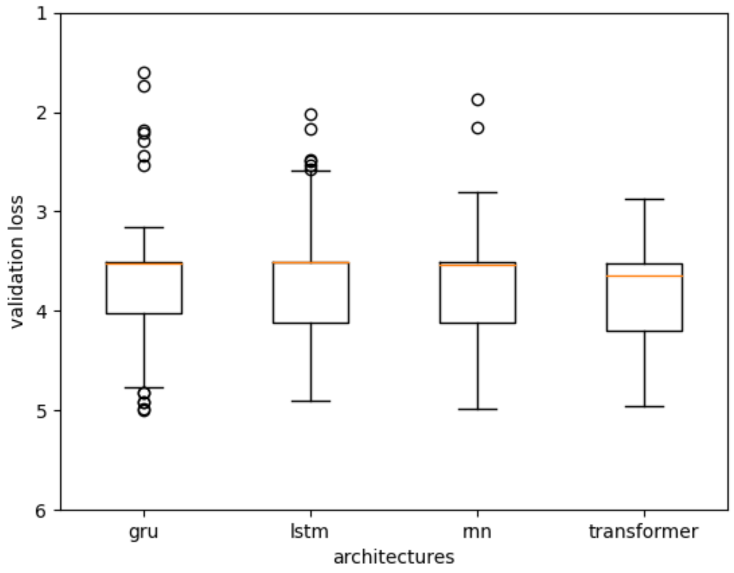

Figure 2. Distribution of optimization results using BOHB without additional information. We optimize over the kernel sizes, stride, number of channels, dropout, and output dimension of the recursive unit and over the number of heads for the attention layer, dropout, the number of linear layers, and embedding dimension when using the transformer-based classification model. We can see a slightly higher mean for the GRU-based models. The best architecture here also belongs to GRU models (see outlier)

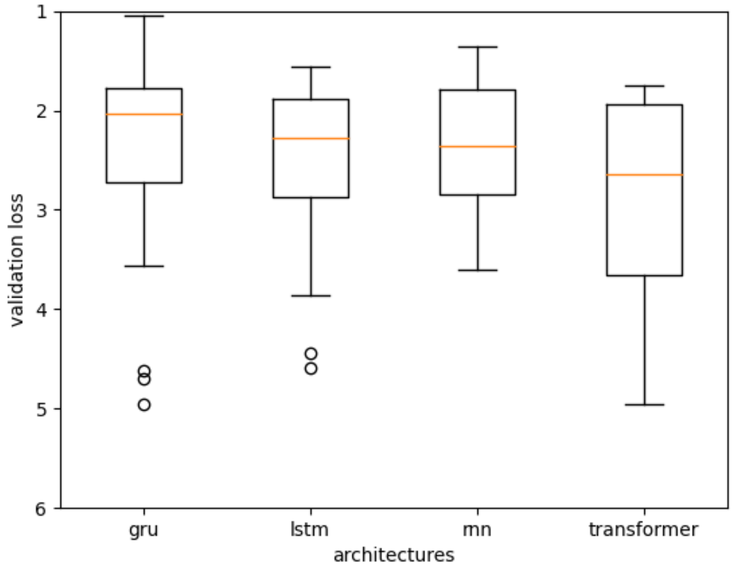

Figure 3.

Optimization with additional information. Here we also optimize over the number of nodes used to process the additional data. The validation loss is lower for all baseline architectures, using additional information compared to the version without additional information. Again GRU based models do perform best.

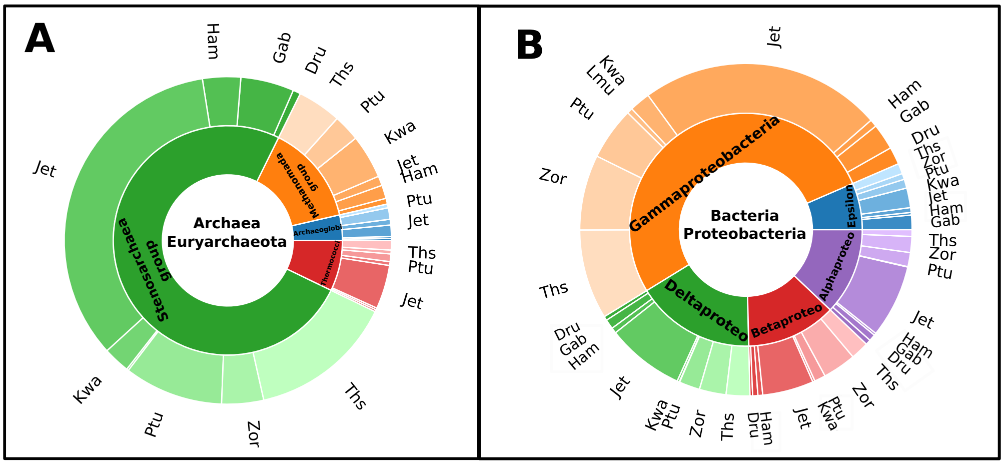

Figure 4. Overview over distribution for phyla Archaea Eurayarchaeota (A) and Bacteria Proteobacteria with associated classes. The outer circle also shows the distribution o the cassettes found in each class

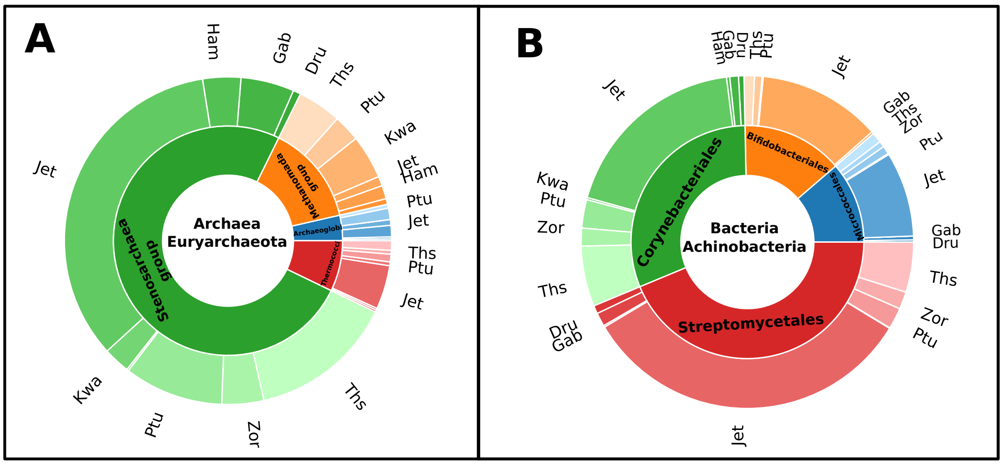

Figure 5. Overview over distribution for phyla Archaea Eurayarchaeota (A) and Bacteria Achinobacteria with associated classes. The outer circle also shows the distribution o the cassettes found in each class
