# Supplementary material for: Deepdefense: annotation of immune systems in prokaryotes using deep learning
Source: Gigascience. 2024 Oct 10;13:giae062. doi: 10.1093/gigascience/giae062 (PMC11959188; doi:10.1093/gigascience/giae062)
Supplement: giae062_Response_to_Reviewer_Comments_Original_Submission [file giae062_response_to_reviewer_comments_original_submission.pdf]

Author's Response To Reviewer Comments

1) Reviewer #1
The paper designed a deep learning based model to identify CRISPR-Cas system. I have some questions as follows.
1. The author reported that they are concerned about the immune system of prokaryotes. What I see here seems to be only targeting the proteins in the system. Do I understand correctly? If only the relevant proteins are predicted, why are the other components in the
system not predicted?

Author’s response
We thank the reviewer for his comment. Our concern is about new immune systems rather than CRISPR-Cas systems. The components of the new immune systems are not well characterized yet.

2. The performance comparison with existing tools is unclear.

Author’s response
Thank you for your comment. Our method is based on a train/test set that is extracted from the literature. For the HMM-based methods, all available proteins are taken and a model is generated without using a train/test split. Hence, our test set would be part of the training set
of the HMMs, and thus a comparison using an independent test set is not possible. We, however, did find another way to compare the tool (see following answer).

3. Why is the model designed by the author better? What is the reason? What features make the model better? What are the drawbacks of the model?

Author’s response
We appreciate the reviewer's point. Current prediction tools rely on Hidden Markov Models (HMMs) constructed from clusters of sequence alignments. The challenge lies in creating accurate clusters, as too much similarity limits predictions, while diversity leads to false
positives. This process demands extensive time and manual intervention. In contrast, deep learning models excel in understanding nuanced features, capturing complex relationships,
and enhancing annotation effectiveness. The second improvement is that we are also detecting cassettes instead of single proteins only, which provides more evidence. To contrast our method with the hmm-based approach, we use sequences with neighboring proteins identified as associated with immune system cassettes and examine the E-value distribution for sequences with reasonably high homology and matching sequence length.
Despite substantial sequence homology and appropriate neighborhood alignment, a noteworthy number of proteins identified by our approach remains unidentified by the existing HMM-based approach (refer to the figure 5 in the paper for further details).
Also, we have clarified the introduction section by adding a paragraph explaining the features that make the deep learning model better.

4. Have some biological mechanisms been discovered or elucidated through this model?

Author’s response
We shared our results with collaborators, who plan to explore systems based on our model.

2) Reviewer #2
Hauns et al. proposed a deep learning method, Deepdefense, for predicting immune systems in prokaryotes. While the method is delicately designed and has the potential to benefit the scientific community, the manuscript requires substantial revisions before
publication.

1. The figures were cited out of order as compared to their appearance in the manuscript, and there are

overlaps between Figures 3 and 5. Please consider reorganizing them for better coherence and adherence to the manuscript's sequence.

Author’s response
We thank the reviewer for his comment. We fixed the order of figures, modified figure so that there is less overlap now.

2. The Results section was difficult to follow, before I read through the Materials and Methods section. Please consider moving some of the key contents, including deep learning framework design and cassette pipeline construction, in the Materials and Methods section to the Results section or a section before the Results section.

Author’s response
Thank you for your comment. We have changed accordingly.

3. Please consider providing more details for the above key contents, as this is the key contribution of the paper in order to identify novel immune systems that are different from CRISPR. And this is also key to understanding why the tables use the average distance as a
major metric to measure the performance of different model
combinations.

Author’s response
We thank the reviewer for his comment. We have expanded and revised the introduction and cassette pipeline sections and added an explanation to the corresponding table.

4. Please consider re-organizing the Data Description section. What is the proportion of training, test, and validation data? How to use a 10-fold CV split to ensure enough training data samples are available for subclasses with few samples? Please make it clear: "We also
create test sets using 10 % of the data with a maximal sequence homology of 95, 90, and 80, respectively."

Author’s response
Thank you for your comment. We have revised it accordingly.

5. Please provide rationales for the selection of cut-off values like ɑ - consider using literature or experiments to support this. In addition, the key parameters of deep learning models should be provided (in the supplementary file) for readers for reproduction purpose.

Author’s response
Thank you for your comment. We have revised the section and added paragraphs to the supplementary material and published code as well.
We follow the original publication by selecting ɑ as 3. This choice functions as an outlier detection measure, as it represents the number of standard deviations a point must be from the mean

6. There are already existing algorithms which authors briefly described in the Introduction section. Please provide more details on the shortcomings or limits of existing methods, and why a new method is needed and how the proposed framework will better solve the problems.

Author’s response
Thank you for your comment. We have revised the introduction section by adding the reason for the need for this framework. We also showed the shortcomings of the existing HMM-based approach by considering cassettes detected with our methods. Despite reasonable homology and additional evidence by neighboring proteins of the same immune system, a lot of them were not identified by the HMM-based approach (see Figure 5). Unlike traditional HMM search our tool also detects cassettes and therefore processes additional information regarding the position of the defense genes.

7. I understand the high thresholds of 95, 90, and 80 on sequence homology were due to the limited

volume of samples of some subclasses, but authors could consider exploring the effect of different threshold values (e.g. 30%; 40%; 50%; 60%; 70%) on models' performance. It would be interesting as well to see how the numbers of samples in cassette classes change across these thresholds.

Author’s response
We thank the reviewer for an interesting point. We ran additional experiments at a lower threshold and added the paragraph to the “Prediction Quality after Optimization” section

8. When compared to the HMM search, Deepdefense predicted much more immune system proteins from bacteria, archea and plasmids and phage. How to validate or confirm the superior performance of Deepdefense? Will Deepdefense bring more false positives than HMM, considering HMM is more likely to retrieve those highly homologous proteins? Would it possible to compare HMM with Deepdefense using the test dataset (as this is a gold standard dataset)? It would be interesting as well to visualize the Deepdefense and/or HMM predicted immune system proteins with those known proteins within a 2D embedding to observe their cluster distributions.

Author’s response
We thank the reviewer for an interesting point. We compared the predicted sequences by Deepdefense and HMM approaches and created a new figure (Fig 5) as already described above, which shows the alignment of newly predicted sequences against our training dataset, as well as the results for the E-value of the corresponding HMM run.
Our method is based on a train/test set that is extracted from the literature. For the HMM-based methods, all available proteins are taken and a model is generated without using a train/test split. Hence, our test set would be part of the training set of the HMMs, and thus a comparison using an independent test set is not possible. However, we are convinced that our cassette-based comparison (so figure 5 mentioned above) is providing a good test set for the HMM-based approach.

9. What is the additional information for protein coding? Please make it clear in the manuscript.

Author’s response
Thank you for your comment. We added the details about the protein features to the supplementary material file.

10. Please use the full names when introducing jargons for the first time in the manuscript, e.g. GRU and BOHB.

Author’s response
Thank you for your comment. We have fixed it.
