# Supplementary material for: Deepdefense: annotation of immune systems in prokaryotes using deep learning
Source: Gigascience. 2024 Oct 10;13:giae062. doi: 10.1093/gigascience/giae062 (PMC11959188; doi:10.1093/gigascience/giae062)
Supplement: giae062_Response_to_Reviewer_Comments_Revision_1 [file giae062_response_to_reviewer_comments_revision_1.pdf]

Author's Response To Reviewer Comments

responses to the revision is included in the main manuscript file.
