# Supplementary material for: Deepdefense: annotation of immune systems in prokaryotes using deep learning
Source: Gigascience. 2024 Oct 10;13:giae062. doi: 10.1093/gigascience/giae062 (PMC11959188; doi:10.1093/gigascience/giae062)
Supplement: giae062_Response_to_Reviewer_Comments_Revision_2 [file giae062_response_to_reviewer_comments_revision_2.pdf]

Author's Response To Reviewer Comments

As suggested by the editor we:

1. included the RRID number where appropriate
2. added a zenodo doi to the manuscript
3. added the RRID citation to the paragraph describing the availability
4. added a final sentence to "Data availability"
5. moved bio.tools and SciCrunch information
6. added "HMM" to the list of abbreviations
7. added the proper GigaDB citation to the references
