# Supplementary material for: Deepdefense: annotation of immune systems in prokaryotes using deep learning
Source: Gigascience. 2024 Oct 10;13:giae062. doi: 10.1093/gigascience/giae062 (PMC11959188; doi:10.1093/gigascience/giae062)
Supplement: giae062_Reviewer_1_Report_Original_Submission — Hao Lin -- 11/21/2023 [file giae062_reviewer_1_report_original_submission.pdf]

Reviewer Report

Title: Deepdefense: Annotation of immune systems in prokaryotes using DeepLearning

Version: Original Submission **Date:** 11/21/2023

Reviewer name: Hao Lin

Reviewer Comments to Author:

The paper designed a deep learning based model to identify CRISPR-Cas system. I have some questions as follows.1. The author reported that they are concerned about the immune system of prokaryotes. What I see here seems to be only targeting the proteins in the system. Do I understand correctly? If only the relevant proteins are predicted, why are the other components in the system not predicted?2. The performance comparison with existing tools is unclear.3. Why is the model designed by the author better? What is the reason? What features make the model better? What are the drawbacks of the model?4. Have some biological mechanisms been discovered or elucidated through this model?

Level of Interest

Please indicate how interesting you found the manuscript: Choose an item.

Quality of Written English

Please indicate the quality of language in the manuscript: Choose an item.

Declaration of Competing Interests

Please complete a declaration of competing interests, considering the following questions:

- Have you in the past five years received reimbursements, fees, funding, or salary from an organisation that may in any way gain or lose financially from the publication of this manuscript, either now or in the future?
- Do you hold any stocks or shares in an organisation that may in any way gain or lose financially from the publication of this manuscript, either now or in the future?
- Do you hold or are you currently applying for any patents relating to the content of the manuscript?
- Have you received reimbursements, fees, funding, or salary from an organization that holds or has applied for patents relating to the content of the manuscript?
- Do you have any other financial competing interests?
- Do you have any non-financial competing interests in relation to this paper?

If you can answer no to all of the above, write 'I declare that I have no competing interests' below. If your reply is yes to any, please give details below.

I declare that I have no competing interests

I agree to the open peer review policy of the journal. I understand that my name will be included on my report to the authors and, if the manuscript is accepted for publication, my named report including any attachments I upload will be posted on the website along with the authors' responses. I agree for my report to be made available under an Open Access Creative Commons CC-BY license (http://creativecommons.org/licenses/by/4.0/). I understand that any comments which I do not wish to be included in my named report can be included as confidential comments to the editors, which will not be published.

Choose an item.

To further support our reviewers, we have joined with Publons, where you can gain additional credit to further highlight your hard work (see: https://publons.com/journal/530/gigascience). On publication of this paper, your review will be automatically added to Publons, you can then choose whether or not to claim your Publons credit. I understand this statement.

Yes Choose an item.
