# Supplementary material for: Deepdefense: annotation of immune systems in prokaryotes using deep learning
Source: Gigascience. 2024 Oct 10;13:giae062. doi: 10.1093/gigascience/giae062 (PMC11959188; doi:10.1093/gigascience/giae062)
Supplement: giae062_Reviewer_2_Report_Original_Submission — Jiawei Wang -- 11/22/2023 [file giae062_reviewer_2_report_original_submission.pdf]

Reviewer Report

Title: Deepdefense: Annotation of immune systems in prokaryotes using DeepLearning

Version: Original Submission **Date:** 11/22/2023

Reviewer name: Jiawei Wang

Reviewer Comments to Author:

Hauns et al. proposed a deep learning method, Deepdefense, for predicting immune systems in prokaryotes. While the method is delicately designed and has the potential to benefit the scientific community, the manuscript requires substantial revisions before publication.1. The figures were cited out of order as compared to their appearance in the manuscript, and there are overlaps between Figures 3 and 5. Please consider reorganizing them for better coherence and adherence to the manuscript's sequence.2. The Results section was difficult to follow, before I read through the Materials and Methods section. Please consider moving some of the key contents, including deep learning framework design and cassette pipeline construction, in the Materials and Methods section to the Results section or a section before the Results section.3. Please consider providing more details for the above key contents, as this is the key contribution of the paper in order to identify novel immune systems that are different from CRISPR. And this is also key to understanding why the tables use the average distance as a major metric to measure the performance of different model combinations.4. Please consider re-organizing the Data Description section. What is the proportion of training, test, and validation data? How to use a 10-fold CV split to ensure enough training data samples are available for subclasses with few samples? Please make it clear: "We also create test sets using 10 % of the data with a maximal sequence homology of 95, 90, and 80, respectively."5. Please provide rationales for the selection of cut-off values like Î± - consider using literature or experiments to support this. In addition, the key parameters of deep learning models should be provided (in the supplementary file) for readers for reproduction purpose.6. There are already existing algorithms which authors briefly described in the Introduction section. Please provide more details on the shortcomings or limits of existing methods, and why a new method is needed and how the proposed framework will better solve the problems.7. I understand the high thresholds of 95, 90, and 80 on sequence homology were due to the limited volume of samples of some subclasses, but authors could consider exploring the effect of different threshold values (e.g. 30%; 40%; 50%; 60%; 70%) on models' performance. It would be interesting as well to see how the numbers of samples in cassette classes change across these thresholds.8. When compared to the HMM search, Deepdefense predicted much more immune system proteins from bacteria, archea and plasmids and phage. How to validate or confirm the superior performance of Deepdefense? Will Deepdefense bring more false positives than HMM, considering HMM is more likely to retrieve those highly homologous proteins? Would it possible to compare HMM with Deepdefense using the test dataset (as this is a gold standard dataset)? It would be interesting as well to visualize the Deepdefense and/or HMM predicted immune system proteins with those known proteins within a 2D embedding to observe their cluster distributions.9. What is the additional information for protein coding? Please make

it clear in the manuscript.10. Please use the full names when introducing jargons for the first time in the manuscript, e.g. GRU and BOHB.

Level of Interest

Please indicate how interesting you found the manuscript: Choose an item.

Quality of Written English

Please indicate the quality of language in the manuscript: Choose an item.

Declaration of Competing Interests

Please complete a declaration of competing interests, considering the following questions:

- Have you in the past five years received reimbursements, fees, funding, or salary from an organisation that may in any way gain or lose financially from the publication of this manuscript, either now or in the future?
- Do you hold any stocks or shares in an organisation that may in any way gain or lose financially from the publication of this manuscript, either now or in the future?
- Do you hold or are you currently applying for any patents relating to the content of the manuscript?
- Have you received reimbursements, fees, funding, or salary from an organization that holds or has applied for patents relating to the content of the manuscript?
- Do you have any other financial competing interests?
- Do you have any non-financial competing interests in relation to this paper?

If you can answer no to all of the above, write 'I declare that I have no competing interests' below. If your reply is yes to any, please give details below.

I declare that I have no competing interests

I agree to the open peer review policy of the journal. I understand that my name will be included on my report to the authors and, if the manuscript is accepted for publication, my named report including any attachments I upload will be posted on the website along with the authors' responses. I agree for my report to be made available under an Open Access Creative Commons CC-BY license (http://creativecommons.org/licenses/by/4.0/). I understand that any comments which I do not wish to be included in my named report can be included as confidential comments to the editors, which will not be published.

Choose an item.

To further support our reviewers, we have joined with Publons, where you can gain additional credit to further highlight your hard work (see: https://publons.com/journal/530/gigascience). On publication of this paper, your review will be automatically added to Publons, you can then choose whether or not to claim your Publons credit. I understand this statement.

Yes Choose an item.
