# Supplementary material for: Deepdefense: annotation of immune systems in prokaryotes using deep learning
Source: Gigascience. 2024 Oct 10;13:giae062. doi: 10.1093/gigascience/giae062 (PMC11959188; doi:10.1093/gigascience/giae062)
Supplement: giae062_Reviewer_2_Report_Revision_1 — Jiawei Wang -- 2/27/2024 [file giae062_reviewer_2_report_revision_1.pdf]

Reviewer Report

Title: Deepdefense: Annotation of immune systems in prokaryotes using DeepLearning

Version: Revision 1 **Date:** 2/27/2024

Reviewer name: Jiawei Wang

Reviewer Comments to Author:

Authors have addressed the major issues I proposed, and here are some minor comments that I hope can help improve the presentation of the manuscript.1. Please make the following statement in the Abstract section clearer:However, these approaches frequently rely on the assumption of a closed world, which is often different in practice.I saw there was a reference for this statement in the Introduction section, but please make it more readable here to readers.2. Please double check the following statement in the Introduction section:As the own DNA is methylated, this protects any unmethylated foreign DNA.3. The following two sentences in the Introduction section are repetitive - please consider re-organising them:We choose to employ deep learning with the expectation that the automatic generation of intricate features will enable us to identify proteins even with lower structural similarity to already known samples. We opt for deep learning, anticipating that its capacity for automatically generating intricate features will enable the identification of proteins, even those with lower structural similarity to known samples.4. In the Introduction section, it says:We create twelve different features characterizing the used proteins (e.g. protein length, instability index) as in [30]. The full list can be found in the supplementary Table S1.But Table S1 only shows 8 protein properties. Please clarify this.5. The title of Figure S3 writes "Additionally to optimization without additional information". "Without" or "With"? Please double check.6. Please consider updating Figures 4, 6 & 7 in high-quality - current versions are hard to read.

Level of Interest

Please indicate how interesting you found the manuscript: Choose an item.

Quality of Written English

Please indicate the quality of language in the manuscript: Choose an item.

Declaration of Competing Interests

Please complete a declaration of competing interests, considering the following questions:

- Have you in the past five years received reimbursements, fees, funding, or salary from an organisation that may in any way gain or lose financially from the publication of this manuscript, either now or in the future?

- Do you hold any stocks or shares in an organisation that may in any way gain or lose financially from the publication of this manuscript, either now or in the future?
- Do you hold or are you currently applying for any patents relating to the content of the manuscript?
- Have you received reimbursements, fees, funding, or salary from an organization that holds or has applied for patents relating to the content of the manuscript?
- Do you have any other financial competing interests?
- Do you have any non-financial competing interests in relation to this paper?

If you can answer no to all of the above, write 'I declare that I have no competing interests' below. If your reply is yes to any, please give details below.

I declare that I have no competing interests

I agree to the open peer review policy of the journal. I understand that my name will be included on my report to the authors and, if the manuscript is accepted for publication, my named report including any attachments I upload will be posted on the website along with the authors' responses. I agree for my report to be made available under an Open Access Creative Commons CC-BY license (http://creativecommons.org/licenses/by/4.0/). I understand that any comments which I do not wish to be included in my named report can be included as confidential comments to the editors, which will not be published.

Choose an item.

To further support our reviewers, we have joined with Publons, where you can gain additional credit to further highlight your hard work (see: https://publons.com/journal/530/gigascience). On publication of this paper, your review will be automatically added to Publons, you can then choose whether or not to claim your Publons credit. I understand this statement.

Yes Choose an item.
